# 4,6-α-Glucanotransferase activity occurs more widespread in *Lactobacillus* strains and constitutes a separate GH70 subfamily

**DOI:** 10.1007/s00253-012-3943-1

**Published:** 2012-02-25

**Authors:** Hans Leemhuis, Willem P. Dijkman, Justyna M. Dobruchowska, Tjaard Pijning, Pieter Grijpstra, Slavko Kralj, Johannis P. Kamerling, Lubbert Dijkhuizen

**Affiliations:** 1 Microbial Physiology, Groningen Biomolecular Sciences and Biotechnology Institute (GBB), University of Groningen, Nijenborgh 7, 9747 AG Groningen, The Netherlands; 2 Biophysical Chemistry, Groningen Biomolecular Sciences and Biotechnology Institute (GBB), University of Groningen, Nijenborgh 7, 9747 AG Groningen, The Netherlands; 3 Carbohydrate Competence Center (CCC), Nijenborgh 7, 9747 AG Groningen, The Netherlands; 4 Present Address: Genencor, Archimedesweg 30, 2333 CN Leiden, The Netherlands

**Keywords:** α-Glucan, Fiber, Glucansucrase, Glycoside hydrolase, 4,6-α-Glucanotransferase, Isomaltooligosaccharide, Starch

## Abstract

Family 70 glycoside hydrolase glucansucrase enzymes exclusively occur in lactic acid bacteria and synthesize a wide range of α-d-glucan (abbreviated as α-glucan) oligo- and polysaccharides. Of the 47 characterized GH70 enzymes, 46 use sucrose as glucose donor. A single GH70 enzyme was recently found to be inactive with sucrose and to utilize maltooligosaccharides [(1→4)-α-d-glucooligosaccharides] as glucose donor substrates for α-glucan synthesis, acting as a 4,6-α-glucanotransferase (4,6-αGT) enzyme. Here, we report the characterization of two further GH70 4,6-αGT enzymes, i.e., from *Lactobacillus reuteri* strains DSM 20016 and ML1, which use maltooligosaccharides as glucose donor. Both enzymes cleave α1→4 glycosidic linkages and add the released glucose moieties one by one to the non-reducing end of growing linear α-glucan chains via α1→6 glycosidic linkages (α1→4 to α1→6 transfer activity). In this way, they convert pure maltooligosaccharide substrates into linear α-glucan product mixtures with about 50% α1→6 glycosidic bonds (isomalto/maltooligosaccharides). These new α-glucan products may provide an exciting type of carbohydrate for the food industry. The results show that 4,6-αGTs occur more widespread in family GH70 and can be considered as a GH70 subfamily. Sequence analysis allowed identification of amino acid residues in acceptor substrate binding subsites +1 and +2, differing between GH70 GTF and 4,6-αGT enzymes.

**Electronic supplementary material:**

The online version of this article (doi:10.1007/s00253-012-3943-1) contains supplementary material, which is available to authorized users.

## Introduction

The glycoside hydrolase (GH) family 70 (Cantarel et al. 2009) is a group of extracellular bacterial glucansucrase (GTF) enzymes that synthesize α-d-glucan (abbreviated as α-glucan) polymers such as dextran, mutan, alternan, and reuteran (Côté and Robyt 1982; Monchois et al. 1999; van Hijum et al. 2006; Moulis et al. 2006; Schwab et al. 2007). These polymers form a protective layer around the cell (Kaditzky et al. 2008; Walter et al. 2008). In theory, four types of α-glycosidic bonds can be formed between d-glucopyranose residues (at C-2, C-3, C-4, and C-6), and all four product specificities have been found among the different GH70 enzymes (Fabre et al. 2005; van Hijum et al. 2006). The GH70 enzymes from lactobacilli typically form products with two types of α-glycosidic linkages and synthesize branches, resulting in complex α-glucan structures. It was generally accepted that all GH70 enzymes use sucrose as glucose donor, but recently, we reported that *Lactobacillus reuteri* 121 encodes a GH70 enzyme, called 4,6-α-glucanotransferase (4,6-αGT-B), that uses maltooligosaccharides (α1→4-linked glucose residues), instead of sucrose, as glucose donor to synthesize linear isomalto/ maltooligosaccharide product mixtures with up to 33% isomalto-sequences (α1→6-linked glucose residues) (Kralj et al. 2011; Dobruchowska et al. 2012). In addition, dextrin dextrinases (EC 2.4.1.2) form isomalto/maltooligosaccharides; however, these products contain branches (Naessens et al. 2005), whereas the 4,6-αGT-B products are linear (Dobruchowska et al. 2012). Although the physiological function of this 4,6-αGT-B enzyme is still unknown, it is interesting to note that this *L. reuteri* 121 also contains a standard type of GH70 enzyme (GTF-A), which synthesizes reuteran (a branched glucan with α1→4 and α1→6 linkages) from sucrose (Kralj et al. 2002; van Leeuwen et al. 2008a). The observation that the recombinant GTF-A enzyme makes an α-glucan polymer indistinguishable from the α-glucan polymer isolated from *L. reuteri* 121 supernatant implies that 4,6-αGT-B has no essential role in the synthesis of the extracellular α-glucan under the conditions tested, e.g., rich media with sucrose.

Today (November 2011), 135 GH70 sequences are reported in the CAZy database (Cantarel et al. 2009), of which 47 have been experimentally characterized (http://www.cazy.org). Three-dimensional structures have been reported for the N-terminally truncated GTF-180 enzyme of *L. reuteri* 180 (Vujičić-Žagar et al. 2010), which makes a branched glucan consisting of α1→6 and α1→3 glycosidic bonds (Kralj et al. 2004a; van Leeuwen et al. 2009), the N- and C-terminally truncated mutan [(1→3)-α-glucan with about 10% α1→6 glycosidic bonds] synthesizer GTF-SI of *Streptococcus mutans* (Ito et al. 2011) and DSR-E of *Leuconostoc mesenteroides* NRRL B1299, which attaches glucose moieties on dextran via α1→2 glycosidic bonds (e.g., branches) (Brison et al. 2012). The catalytic domain of these enzymes has a (β/α)_8_-fold, which is circularly permutated compared to the arrangement of the catalytic domain in the evolutionary related enzymes of the GH13 and GH77 families. Note that also the GH13 amylosucrase enzymes synthesize α-glucans from sucrose (Potocki de Montalk et al. 2000), though only linear (1→4)-α-glucans, whereas all other GH13 enzymes either hydrolyze or disproportionate α-glycosidic bonds between two glucose moieties (Stam et al. 2006; Kelly et al. 2009b). In the GH70 family, as well as in the GH13 and GH77 families, the catalytic nucleophile (Asp1025; numbered according to GTF-180), acid/base (Glu1063), and transition state stabilizer (D1136) are located at the C-terminal ends of the β-strands, as revealed by a sucrose bound structure of GTF-180 (Vujičić-Žagar et al. 2010). Despite the availability of crystal structures and the observation that mutations near the active site can alter the ratio of α-glycosidic bonds synthesized (Hellmuth et al. 2008), the reaction specificity of GH70 is not well understood, limiting the design of GH70 variants capable of synthesizing predefined α-glucan polymers.

Here, we describe the cloning, expression, and characterization of the second and third GH70 enzyme inactive with sucrose as donor substrate. The two GH70 genes of *L. reuteri* DSM20016 and *L. reuteri* ML1 encode enzymes that utilize maltooligosaccharides as substrate to synthesize linear α-glucans with α1→6-linked glucose segments at the non-reducing end of α1→4-linked glucose segments.

## Materials and methods

### Cloning of the *gtfW* gene

A truncated version of the 4,6-αGT-W encoding *gtfW* gene (GenBank accession number ABQ83597) was amplified from the genomic DNA of *L. reuteri* DSM 20016 using the primers For (*Nco*I) 5′-GAT GCA TCC ATG GGC ATA GAT GGT AAG AAC TAC CAC TTC GC-3′ and Rev (*Bam*HI) 5′-ATA TCG ATG GAT CCT ATT AGT GAT GGT GAT GGT GAT GAA TAT TTT CTT GGT TTG CAT AGT AAT CTG C-3′ and high fidelity DNA polymerase (Fermentas), and cloned in pET15b (Novagen), yielding pET15b_4,6-αGT-W. This N-terminally truncated version of 4,6-αGT-W (amino acids 458–1363) carries a fused (His)_6_-tag at its C-terminal. The removal of the N-terminal variable region is based on the cloning and expression of other GTFs, as this improves protein expression without affecting the catalytic properties (Kralj et al. 2004b).

### Cloning of the *gtfML4* gene

Previously, the 3′-end fragment of the 4,6-αGT-ML4 encoding *gtfML4* gene (GeneBank accession number AAU08003.1) has been identified in *L. reuteri* ML1, located upstream of a glucansucrase gene (*gtfML1*). The 5′-end of the *gtfML4* gene was obtained by inverse PCR. Briefly, genomic DNA of *L. reuteri* ML1 was digested by *Bcu*I or *Nsi*I, and the products were ligated at a concentration of ~10 ng/μl using T4 DNA ligase to obtain circular fragments. The ligation mixtures served as PCR template in an inverse PCR with the oligonucleotides HL115, 5′-TGA TCG TCC AGA TGT AGC-3′ and HL116, 5′-CCA GTT ACT TTC ATA GAG G-3′. This yielded PCR fragments of 2 kbp (*Bcu*I) and 8 kbp (*Nsi*I), which were cloned in the pCR-XL-TOPO vector (Invitrogen). The obtained plasmids were used for DNA sequencing, yielding the 5′-end of the *gtfML4* gene.

Based on the DNA sequence obtained with the inverse PCR, the oligonucleotides HL117 (forward, *Nco*I), 5′-TTT TCC ATG GGG AAC CGC GTT GAT TAC TGG-3′ and HL118 (reverse, *Bgl*II), 5′-AAA AGA TCT TAG TGA TGG TGA TGG TGA TGG TTG TTA AAA TTT AAT GAA ATT GC-3′ were designed and used to amplify the gene from chromosomal DNA. The obtained fragment was cloned in pET15b (Novagen), yielding pET15b_4,6-αGT-ML4. This vector encodes an N-terminally truncated version of 4,6-αGT-ML4 (amino acids 714–1620) and carries a fused (His)_6_-tag at its C terminus. Removal of the N-terminal variable region previously was shown to improve GTF protein expression without affecting catalytic properties (Kralj et al. 2004b).

### Expression and purification of 4,6-αGT-W

The 4,6-αGT-W protein was produced in *Escherichia coli* BL21(DE3)/pET15b_4,6-αGT-W cultivated in Luria broth containing 100 mg/l ampicillin. Protein expression was induced at an OD_600_ of 0.4–0.5 by adding isopropyl β-d-1-thiogalactopyranoside to 0.1 mM, and cultivation was continued for 4 h at 30°C. Cells were harvested by centrifugation, washed in phosphate buffer (50 mM, pH 8.0) and resuspended in phosphate buffer (50 mM, pH 8.0), containing NaCl (250 mM), imidazole (10 mM), and β-mercaptoethanol (5 mM). Cell-free extracts were made by sonication followed by centrifugation (10,000×*g*, 1 h). The 4,6-αGT-W protein was purified by nickel affinity chromatography using an 1-ml Hitrap IMAC HP column (GE Healthcare) and a gradient of 10–200 mM imidazole in phosphate buffer (50 mM, pH 8.0), containing NaCl (250 mM). Fractions containing 4,6-αGT-W were pooled. The imidazole and NaCl were removed using a 5-ml Hitrap desalting column (GE Healthcare) run with sodium phosphate buffer (50 mM, pH 8.0), and the protein was loaded on a 1-ml Resource-Q column (GE Healthcare). Protein was eluted with a gradient of 0–1 M NaCl in phosphate buffer (50 mM, pH 8.0). Fractions containing 4,6-αGT-W were transferred to sodium acetate buffer (50 mM, pH 5.5) using a 5-ml Hitrap desalting column. Protein purity was assessed on 8% sodium dodecyl sulfate polyacrylamide gel electrophoresis (SDS-PAGE) and protein concentration measured with Bradford reagent (BioRad) and bovine serum albumin as standard.

### Expression and purification of 4,6-αGT-ML4

The 4,6-αGT-ML4 protein was produced in *E. coli* BL21(DE3)/pET15b_4,6-αGT-ML4 cultivated in Luria broth containing 100 mg/l ampicillin. Protein expression was induced at an OD_600_ of 0.4–0.5 by adding isopropyl β-d-1-thiogalactopyranoside to 0.1 mM, and cultivation was continued for 4 h at 30°C or 24 h at 18°C. Cells were harvested by centrifugation, washed in Tris/HCl buffer (20 mM, pH 8.0), and resuspended in 10 ml BPer lysis reagent (Pierce), followed by centrifugation (10,000×*g*, 15 min). The 4,6-αGT-ML4 protein was purified from inclusion bodies by washing the pellet once with 5 ml BPer plus 2 mg lysozyme for 10 min at room temperature, followed by four washes with 50 ml 10 times diluted BPer. Finally, the inclusion bodies were resuspended in 10 ml 8 M urea, 20 mM Tris/HCl (pH 8.0), 1 mM CaCl_2_, and 1 mM DTT to obtain unfolded protein. Refolding was done by dialysis against 20 mM Tris/HCl (pH 8.0), 1 mM CaCl_2_, and 1 mM dithiothreitol. The 4,6-αGT**-**ML4 protein was then further purified by nickel affinity chromatography using a 1-ml Hitrap IMAC HP column (GE Healthcare). Following washing with 10 mM imidazole in 20 mM Tris/HCl (pH 8.0) with 1 mM CaCl_2_, the protein was eluted with 200 mM imidazole in the same buffer. The imidazole was then removed using a 5-ml Hitrap desalting column (GE Healthcare) run with 20 mM Tris/HCl (pH 8.0) and 1 mM CaCl_2_. Protein purity was assessed on 8% SDS-PAGE and protein concentration measured with Bradford reagent (BioRad) and bovine serum albumin as standard.

### Enzyme assays

All reactions were performed in sodium acetate buffer (25 mM, pH 4.7) with CaCl_2_ (1 mM) at 37°C, unless stated otherwise. The substrate spectra of the enzymes were investigated by incubating the various oligosaccharides (10 mg, 100 mM; Sigma-Aldrich) with 25 μg/ml enzyme. The progress of the reactions was followed by high-pH anion-exchange chromatography (HPAEC) (described below) and/or thin-layer chromatography (TLC) using silica gel 60F254 plates (Merck) run with butanol/ethanol/water (5:3:3, *v*/*v*/*v*). TLC plates were developed with 10% H_2_SO_4_ in methanol/water (1:1, *v*/*v*), followed by heating at 100°C.

For 4,6-αGT-W activity, the optimal pH and temperature were determined over the pH range of 3.5–6.5 (in 25 mM sodium acetate buffer) and the temperature range of 20–60°C, using 100 mM maltose as substrate by following the release of glucose in time using the GOPOP kit (Megazyme) (Kaper et al. 2007). This assay could not be used with 4,6-αGT-ML4 as it is hardly active with maltose. The optimal pH and temperature of 4,6-αGT-ML4 were therefore determined using 50 mM maltotetraose as substrate and by following appearance of products on TLC plates.

The thermal inactivation rate of 4,6-αGT-W was determined by incubating the enzyme at a concentration of 0.26 mg/ml in sodium acetate buffer (50 mM, pH 5.5) with CaCl_2_ (1 mM) at temperatures from 20°C to 55°C in a water bath for 10 min. Samples were then cooled in an ice bath, and residual activity was measured by following the release of glucose using maltose as substrate. The *T*_50_ is defined as the temperature at which 50% of the initial enzyme activity is retained after 10-min incubation.

Kinetic properties of 4,6-αGT-W were determined with maltose (0–500 mM) as substrate and by following the release of glucose. Reactions were initiated by the addition of 4,6-αGT-W enzyme at a concentration of 64 nM. The rates were fitted to the Michaelis–Menten equation.

### Isolation of individual products made by 4,6-αGT-W via HPAEC

Twenty milligrams of maltose was incubated with 16 μg 4,6-αGT-W in 1.17 ml sodium acetate buffer (50 mM, pH 4.7) at 37°C for 4 days. The reaction mixture was freeze-dried, dissolved in 200 μl Milli-Q water, and 20-μl aliquots were loaded on a 9 × 250 mm CarboPac PA-1 column (Dionex), which was run with a gradient of 0–600 mM sodium acetate in 100 mM NaOH (3 ml/min). Eluting peaks were collected manually and neutralized immediately with 4 M acetic acid. The collected fractions were desalted on 150 mg CarboGraph SPE columns (DiscoverySciences) using acetonitrile/water = 1:3 (*v*/*v*) as eluent, followed by lyophilization. The desalted fractions were analyzed by matrix-assisted laser-desorption ionization time-of-flight mass spectrometry (MALDI-TOF-MS) and ^1^H nuclear magnetic resonance (NMR) spectroscopy. Fractions containing multiple oligosaccharides were separated again by HPAEC using a different elution gradient.

### 4,6-αGT-W and 4,6-αGT-ML4 product analysis with hydrolytic enzymes

Reaction products (250 μg) of the enzyme incubations with maltose up to maltoheptaose were dissolved in 10 μl sodium acetate buffer (50 mM) and incubated for 1 day at 37°C with α-amylase (porcine pancreas type I-A, pH 6.5; SigmaAldrich), amyloglucosidase (*Rhizopus*, pH 5.0; Megazyme), or pullulanase M1 (*Klebsiella planticola*, pH 5.0; Megazyme). α-Amylase cleaves α1**→**4 glycosidic bonds in longer α1**→**4 fragments, amyloglucosidase is an exo-acting enzyme that cleaves terminal α1**→**4 and α1**→**6 glycosidic bonds at the non-reducing end to form glucose, and pullulanase M1 cleaves α1**→**6 glycosidic bonds at the reducing end of an α1**→**4 glycosidic bond [in (-)α-d-Glc*p*-(1**→**4)-α-d-Glc*p*-(1**→**6)- sequences] (Domań-Pytka and Bardowski 2004). The degree of degradation was assessed by TLC and/or HPAEC analysis.

### High-pH anion-exchange chromatography

Carbohydrate samples were analyzed on a 4 × 250 mm CarboPac PA-1 column using a Dionex DX500 workstation (Dionex), run with a gradient of 30–600 mM sodium acetate in 100 mM NaOH (1 ml/min), and detected with an ED40 pulsed amperometric detector. Calibration was done by running samples with known concentrations of glucose and maltotetraose.

### Matrix-assisted laser-desorption ionization time-of-flight mass spectrometry

MALDI-TOF-MS experiments were performed on an Axima™ mass spectrometer (Shimadzu), equipped with a nitrogen laser (337 nm, 3 ns pulse width). Positive-ion mode spectra were recorded using the reflector mode at a resolution of 5000 FWHM and delayed extraction (450 ns). The accelerating voltage was 19 kV with a grid voltage of 75.2%; the mirror voltage ratio was 1.12, and the acquisition mass range was 200–3,000 Da. Samples (1 μl) were mixed in 1:1 ratio with 10 mg/ml 2,5-dihydroxybenzoic acid in acetonitrile/water = 1:1 (*v*/*v*). The compounds fly as sodium ion adduct.

### Linkage analysis

Samples (~2 mg) were permethylated using CH_3_I and solid NaOH in Me_2_SO as described previously (Ciucanu and Kerek 1984). After hydrolysis with 2 M trifluoroacetic acid (2 h, 120°C), the partially methylated monosaccharides were reduced with NaBD_4_ (2 h at room temperature, aqueous solution). Conventional work-up, comprising neutralization (by adding 4 M acetic acid) and removal of boric acid by co-evaporation with methanol, followed by acetylation with pyridine/acetic anhydride (1:1, *v*/*v*) (30 min, 120°C), yielded mixtures of partially methylated alditol acetates, which were analyzed by gas-liquid chromatography electron impact mass spectrometry.

### NMR spectroscopy

Resolution-enhanced 1D 500-MHz ^1^H NMR spectra were recorded in D_2_O on a Varian Inova Spectrometer (NMR Center, University of Groningen) or a Bruker DRX-500 spectrometer (Bijvoet Center, Department of NMR Spectroscopy, Utrecht University) at probe temperatures of 300 K. Prior to analysis, samples were exchanged twice in D_2_O (99.9 atm% D, Cambridge Isotope Laboratories, Inc.) with intermediate lyophilization and then dissolved in 0.6 ml D_2_O. Suppression of the HOD signal (on the Bruker DRX-500 spectrometer only) was achieved by applying a WEFT pulse sequence. Chemical shifts (*δ*) are expressed in parts per million by reference to internal acetone (*δ* 2.225 for ^1^H).

### Sequence alignments

The amino acid sequences of GH70 proteins were compared using ClustalW2 run at EMBL-EBI (http://www.ebi.ac.uk) (Larkin et al. 2007; Goujon et al. 2010).

## Results

### Cloning of the *gtfW* and *gtfML4* genes

*L. reuteri* DSM 20016 possesses a *gtfW* gene (4092 bps; GeneBank accession ABQ83597), which encodes a 154-kDa protein, designated 4,6-αGT-W. Sequence comparison indicates that, in analogy with other GH70 enzymes, 4,6-αGT-W possesses a variable N-terminal region (amino acids 1-427), a catalytic domain [amino acids 428–1223; e.g., domains A, B, and C in the crystal structure of GTFs (Vujičić-Žagar et al. 2010; Ito et al. 2011)] and a C-terminal region (amino acids 1224–1363). The signal sequence predictor SignalP 3.0 does not predict an N-terminal signal sequence for 4,6-αGT-W. The enzyme shares 47% sequence identity with 4,6-αGT-B of *L. reuteri* 121; the similarity of domains A, B, and C is much higher with 70% sequence identity. A protein BLAST search identifies three hypothetical GH70 enzymes in the genomes of *L. reuteri* JCM 1112 (Morita et al. 2008), *L. reuteri* MM2-3 (UniProt accession C0YXW9) and *L. reuteri* MM4-1A (UniProt accession C2F8B9) that are virtually identical to 4,6-αGT-W, the only difference being that the three hypothetical proteins have an extra 125 amino acids at their N termini. The next hit in the BLAST search is a hypothetical GH70 protein (UniProt accession C0X0D3) from *Lactobacillus fermentum* ATCC 14931 with 71% sequence identity to 4,6-αGT-W.

Previously, it was shown that *L. reuteri* ML1 possesses a *gtfML1* gene, which encodes a glucansucrase and that the direct upstream DNA sequence shows similarity to GH70 genes (Kralj et al. 2004a), though a full gene sequence was not obtained. This gene fragment was designated as (part of) *gtfML4*, and the encoded protein fragment showed most sequence similarity to 4,6-αGT-B of *L. reuteri* 121. Therefore, we isolated the full *gtfML4* gene by inverse PCR. The gene is 4863 nucleotides and encodes a protein of 180 kDa, which has the typical arrangement of a GH70 family enzyme, with an N-terminal signal sequence of 39 amino acids as predicted by the SignalP 3.0 (Bendtsen et al. 2004), a large variable N-terminal region (amino acids 40–738), a catalytic domain (amino acids 739–1486), and a C-terminal region (amino acids 1487–1620). 4,6-αGT-ML4 shares 96% sequence identity with GTF-106B of *L. reuteri* TMW1.106, which has been reported to slowly hydrolyze sucrose but does not synthesize a polymer from sucrose (Kaditzky et al. 2008) and 86% and 46% sequence identity with 4,6-αGT-B and 4,6-αGT-W, respectively.

**Fig. 1:**
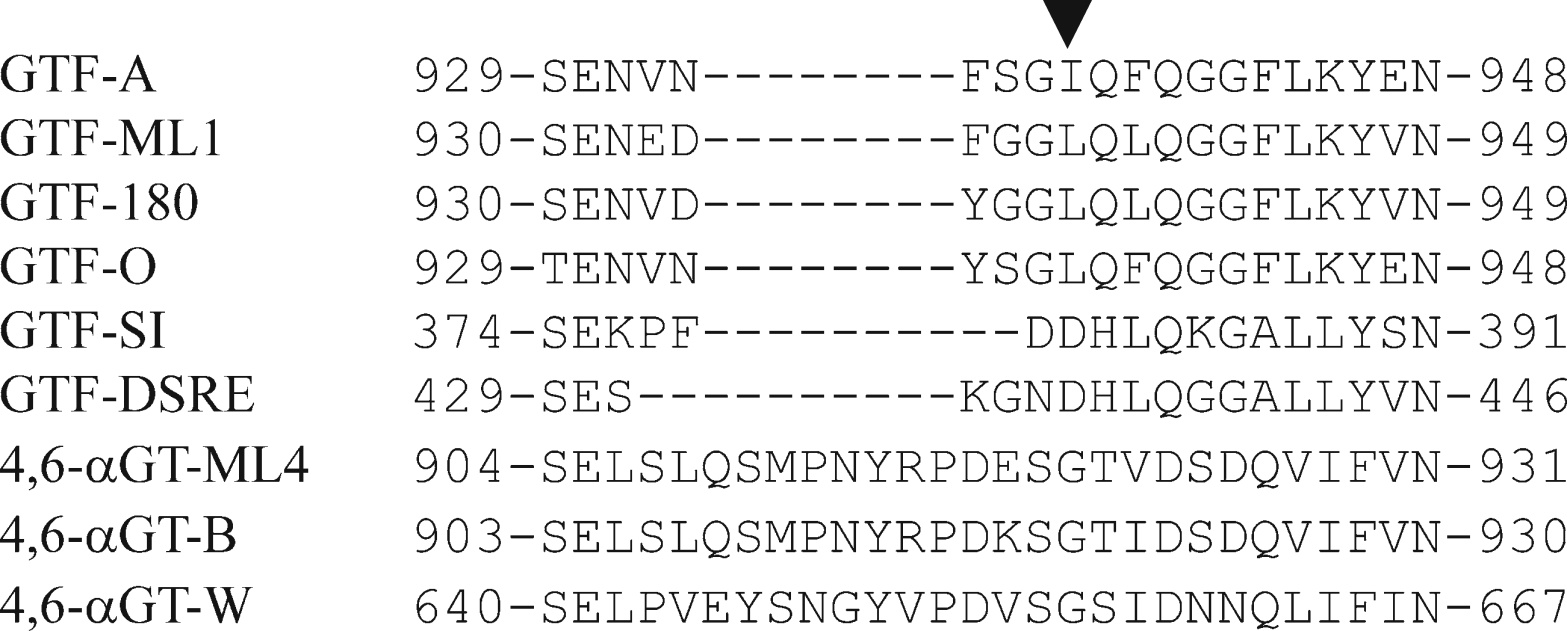
The loop connecting the domains IV and B is longer in 4,6-αGTs then in GTFs. The sequence alignment shows the region around the subsite +1 residue L938 (indicated by the *triangle*) of GTF-180. The sequences used are as follows: GTF-A (Q5SBL9) of *Lactobacillus reuteri* 121; GTF-180 (Q5SBN3) of *L. reuteri* 180; GTF-O (Q4JLC7) of *L. reuteri* ATCC 55730; GTF-ML1 (Q5SBN0) of *L. reuteri* ML1; GTF-DSRE (Q8G9Q2) of *Leuconostoc mesenteroides* NRRL B-1299; GTF-SI (P13470) of *Streptococcus mutans* GS-5; 4,6-αGT-B (Q5SBM0) of *L. reuteri* 121; 4,6-αGT-W (A5VL73) of *L. reuteri* DSM20016; 4,6-αGT-ML4 (Q5SBN1) of *L. reuteri* ML1

Sequence alignments of the GH70 enzymes revealed differences in the amino acids forming the acceptor subsites +1/+2 between 4,6-αGTs and GTFs (Table 1). In addition, it showed that the loop connecting domains IV and B (residues 932-943 in GTF-180), containing the subsite +1 residue 938, is eight residues longer in 4,6-αGTs compared to GTFs (Fig. 1). Phylogenetic analysis of the GH70 protein sequences revealed that the 4,6-αGT proteins form a small but clearly separate cluster (Suppl. Info. Fig. S1).

**Table 1:** Comparison of the amino acid residues at the acceptor subsites +1 and +2 of GH70 enzymes

	GTFs	4,6-αGTs	Function in GTFs
Subsite +1			
938^a^	D,*L*,I^b,c^	G	4,6-αGTs have a longer loop here
981	*L*	L	–
1028	*D*	D	–
1029	*N*	N	–
1065	*W*,G	Y	–
1140	*Q*	N	Glycosidic bond specificity^d^
Subsite +2			
978	Y,F,*A*,Q,S	P	Loop region with variation in length
1028	*D*	D	–
1065	*W*,G	Y	–
1088	*R*,H,K,T,S	F	–
1137	*S*,N	Q	Glycosidic bond specificity^d,e^
1141	T,*D*,S,A,G	L,V	Glycosidic bond specificity^f^

The information is based on the crystal structure of GTF-180 of *L. reuteri* 180 [protein databank files 3HZ3 and 3KLL (Vujičić-Žagar et al. 2010)], and the amino acids are numbered according to GTF-180. Eighty-eight GH70 sequences were used for the alignment, including the three 4,6-αGTs (B, W, and ML4) and the two putative 4,6-αGTs of *Lactobacillus reuteri* JCM 1112 (Uniprot accession number B2G8K2) and *Lactobacillus reuteri* TMW1.106 (Uniprot accession number A9Q0J0). The equivalent residues in the 4,6-αGTs are based on sequence alignments. Catalytic residues are excluded from the comparison as they are conserved in both GTFs and 4,6-αGTs

^a^Amino acids are numbered according to GTF-180 of *L. reuteri* 180 of which the three dimensional structure is known (Vujičić-Žagar et al. 2010)

^b^The amino acids are given in a descending order of occurrence

^c^The residues in GTF-180 are indicated in italics

^d^van Leeuwen et al. (2009)

^e^Irague et al. (2011)

^f^Shimamura et al. (1994)

### Expression and basic properties of 4,6-αGT-W and 4,6-αGT-ML4

N-terminally truncated versions of 4,6-αGT-W (amino acids 458–1363) and 4,6-αGT-ML4 (amino acids 714–1620) were expressed in *E. coli*. 4,6-αGT-W was purified by His-tag affinity and anion-exchange chromatography, yielding 1.0 mg of pure protein per liter of culture. The enzyme displays optimal activity around pH 4.5 and 40–45°C and is resistant to thermal inactivation up to 45°C in sodium acetate buffer (pH 5.5), but at 51°C, it loses half of its activity in 10 min. 4,6-αGT-ML4 was purified from inclusion bodies using urea unfolding, refolding by dialysis and subsequent His-tag affinity chromatography, yielding 1.2 mg of soluble and pure 4,6-αGT-ML4 protein per liter of culture. The enzyme displays optimal activity around pH 4.5 and 40°C.

### 4,6-αGT-W and 4,6-αGT-ML4 disproportionate maltooligosaccharides

**Fig. 2:**
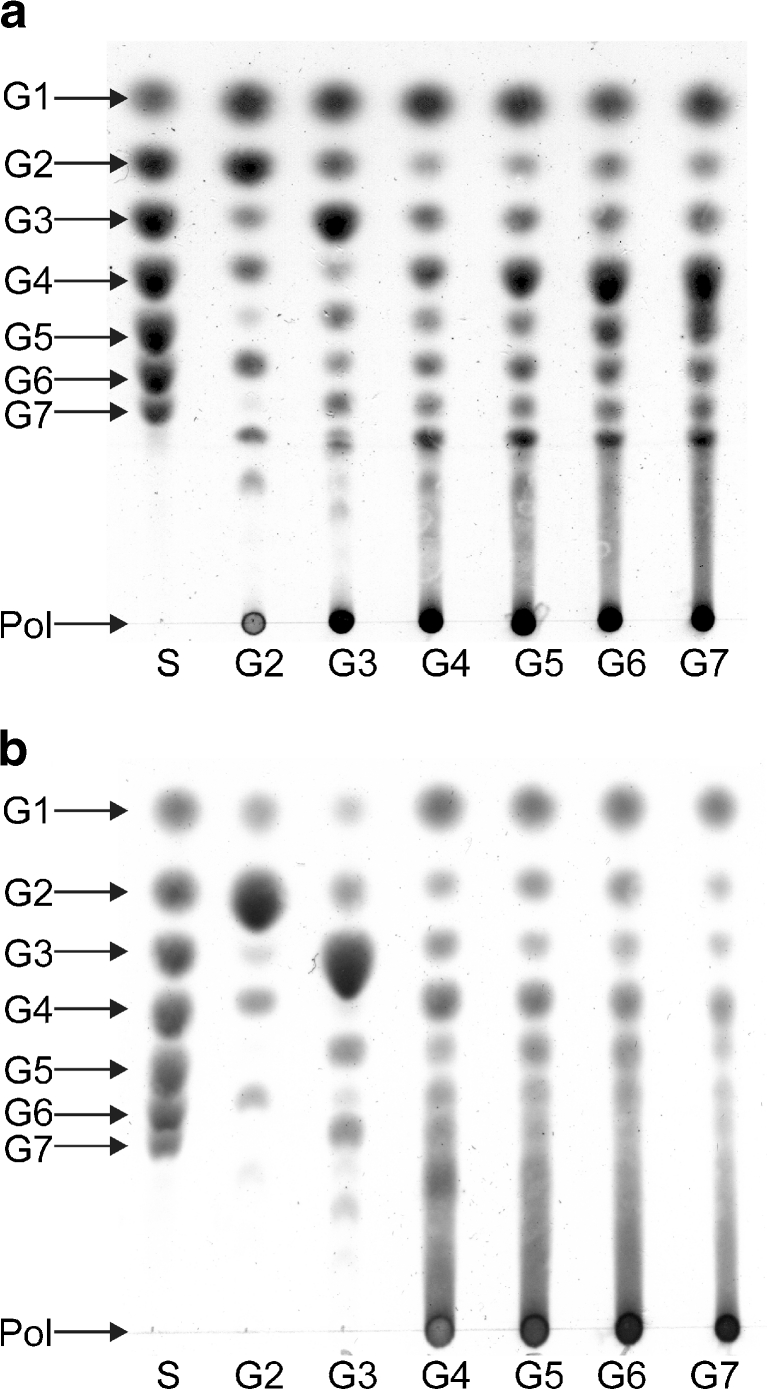
4,6-αGT-W and 4,6-αGT-ML4 disproportionate maltooligosaccharides [(1→4)-α-d-glucooligosaccharides]. TLC analysis of product mixtures from maltooligosaccharide incubations with **a** 4,6-αGT-W and **b** 4,6-αGT-ML4. Conditions: pH 4.7 and 37°C, 40 h, 25 μg/ml enzyme. *S* standard: glucose (G1) to maltoheptaose (G7); *Pol* polymer; *lanes 2–7* are the product mixtures obtained from maltose (G2) to maltoheptaose (G7)

The substrate preference of the 4,6-αGT-W and 4,6-αGT-ML4 enzymes was explored by incubating them with various oligosaccharides. As shown by TLC, both enzymes use linear maltooligosaccharides [(1→4)-α-d-glucooligosaccharides] as substrate, forming a range of shorter and longer products (Fig. 2), but not sucrose (the typical substrate of GH70 enzymes), trehalose, raffinose, 1-kestose, nystose, isomaltose, and isomaltopentaose. The difference between the two enzymes is that 4,6-αGT-W already efficiently disproportionates the disaccharide maltose, whereas 4,6-αGT-ML4 requires maltotetraose or longer maltooligosaccharides (Fig. 2). Besides oligomeric material, TLC analysis showed that both enzymes also produce polymeric material [degree of polymerisation (DP) >10] (Fig. 2). Note that the polymeric material is not of very high molecular weight. For the homolog enzyme 4,6-αGT-B, we showed that the larger molecules synthesized from maltoheptaose had DPs below 50 (Dobruchowska et al. 2012).

The initial rate of 4,6-αGT-W with maltose was determined by measuring the release of glucose (as maltose + maltose→a DP3 compound + glucose) using glucose oxidase, displaying a *k*_cat_ of 37 ± 4 s^−1^ and a *K*_M_ of 150 ± 40 mM. The enzyme also processes maltopentaose, yielding maltotetraose + glucose (hydrolysis) or maltotetraose + a DP6 compound (transglycosylation). With 43 mM of maltopentaose as substrate, the initial rate of glucose formation was 56% of that of maltotetraose formation, showing that the enzyme is rather hydrolytic at the start of the reaction. However, when reaction products start to accumulate, transglycosylation becomes more efficient, as the reaction products are better acceptor substrates than maltopentaose.

### 4,6-αGT-W and 4,6-αGT-ML4 form α1→6 glycosidic bonds

HPAEC analysis of the reaction products of the incubations of 4,6-αGT-W and 4,6-αGT-ML4 with maltooligosaccharides [(1→4)-α-d-glucooligosaccharides] of DP2 to DP7 (for DP7, see Suppl. Info. Fig. S2) revealed the formation of products with retention times different from those of maltooligosaccharides. The elution profiles of both enzyme incubations per DP also showed that the product ensembles are not identical, despite the fact that both reaction mixtures contain the same type of glycosidic linkages, at nearly identical ratios, as discussed below.

**Fig. 3:**
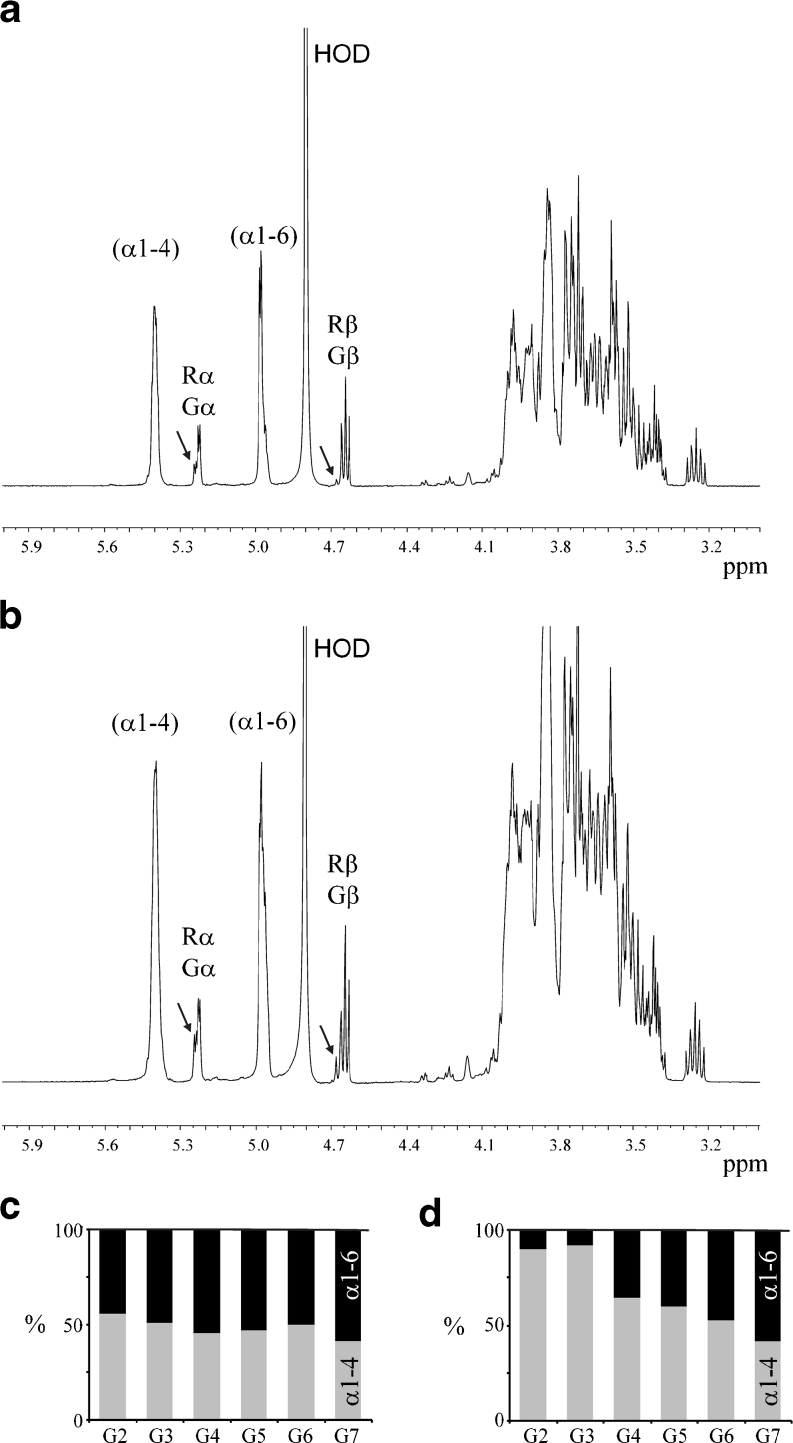
4,6-αGT-W and 4,6-αGT-ML4 form α1→6 glycosidic linkages. 500-MHz ^1^H NMR spectra (without water suppression) of product mixtures obtained from maltohexaose (DP6) incubations with **a** 4,6-αGT-W or **b** 4,6-αGT-ML4. The *bar diagrams* give the percentage of α1→4 (*light gray*) and α1→6 glycosidic linkages (*black*) in the product mixtures obtained from maltose (G2) to maltoheptaose (G7) incubated with **c** 4,6-αGT-W or **d** 4,6-αGT-ML4. The anomeric signals reflecting the α1→4 and α1→6 glycosidic linkages are indicated by (α1-4) and (α1-6), respectively. The anomeric signals assigned as Gα/β and Rα/β indicate the presence of free glucose and the presence of reducing -(1→4)-d-Glc*p* units, respectively. The *arrows* in the spectra of the product mixtures indicate the presence of small amounts of reducing -(1→6)-d-Glc*p* units

^1^H NMR analysis of the various product mixtures obtained with 4,6-αGT-W and 4,6-αGT-ML4 showed the presence of α1→4 linkages (H-1, *δ* ~5.40) and newly formed α1→6 linkages (H-1, *δ* ~4.97; broad signal). As typical examples, Fig. 3 presents the very similar ^1^H NMR spectra for the product mixtures generated from maltohexaose, showing a linkage ratio α1→4:α1→6 = 50:50 for 4,6-αGT-W (Fig. 3a) and a linkage ratio α1→4:α1→6 = 53:47 for 4,6-αGT-ML4 (Fig. 3b). The slightly different built-up of the broad H-1 signal at *δ* ~4.97, comparing both cases, supports the HPAEC observation that different product ensembles are formed. The reducing-end glucose residues are 4-substituted [-(1→4)-d-Glc*p*; Rα H-1, *δ* 5.225; Rβ H-1, *δ* 4.652)]. The spectra also revealed the presence of free glucose (Gα H-1, *δ* 5.225; Gβ H-1, *δ* 4.637). Furthermore, small amounts of six-substituted reducing-end glucose residues [-(1→6)-d-Glc*p*; Rα H-1, *δ* 5.241; Rβ H-1, *δ* 4.670) were detected, demonstrating that both enzymes can use glucose as acceptor to make isomaltose, which can then be further elongated by the enzyme. The ^1^H NMR spectra (Fig. 3) reveal that both 4,6-αGT-W and 4,6-αGT-ML4 create linear structures only, as no signal typical for -(1→4,6)-α-d-Glc*p*-(1→4)- branches is seen [H-1, *δ* ~5.36)] (van Leeuwen et al. 2008a).

For the conversion of maltotetraose by 4,6-αGT-W (Suppl. Info. Fig. S3), methylation analysis supports the conclusion that only 4-O- and 6-O-substituted glucose residues are present, but no branched residues, thereby demonstrating that indeed the products are linear. Interestingly, the ^1^H NMR analysis revealed that in case of 4,6-αGT-W the size of the substrate had only little influence on the α1→4:α1→6 glycosidic linkage ratio in the product mixtures (Fig. 3c), whereas for 4,6-αGT-ML4 the percentage of α1→6 glycosidic linkages introduced increased with increasing size of the substrate (Fig. 3d). In conclusion, both 4,6-αGT-W and 4,6-αGT-ML4 convert (1→4)-α-d-glucooligosaccharides in linear α-glucans with α1→4 and α1→6 glycosidic linkages.

### Purification and characterization of 4,6-αGT-W products

**Fig. 4:**
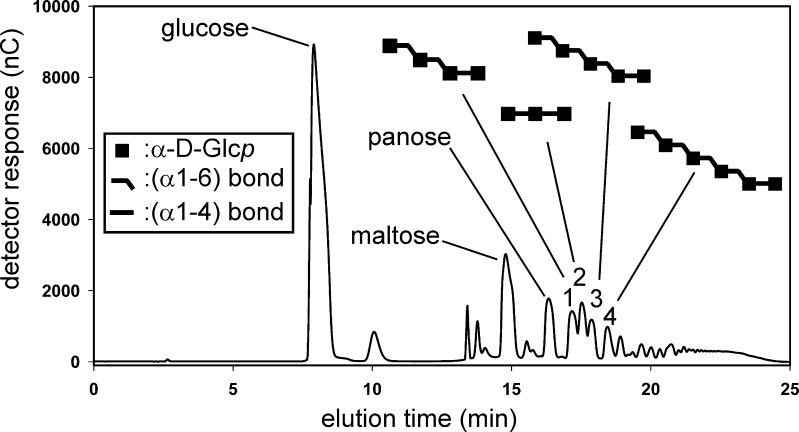
HPAEC elution profile of the reaction mixture obtained from maltose, incubated with 4,6-αGT-W at pH 4.7 and 37°C for 4 days. Fractions 1–4 were collected, and after desalting, the products were characterized by ^1^H NMR spectroscopy (Suppl. Info. Fig. 4a–d), making use of a ^1^H NMR library data base of α-glucans (van Leeuwen et al. 2008b; Dobruchowska et al. 2012) (and references cited therein). Identified structures are indicated in *symbol notation above the peaks*; for the complete names, see text

To gain more insight in the carbohydrate structures made by 4,6-αGT-W, the reaction mixture obtained from maltose was separated by HPAEC (Fig. 4). The isolated compounds were analyzed by MALDI-TOF-MS for their molecular mass and for their purity in terms of DP. Fractions containing multiple oligosaccharides were separated again by HPAEC using a different elution gradient. The structures of the isolated compounds were established by 1D ^1^H NMR spectroscopy making use of a ^1^H NMR library data base of α-glucans (van Leeuwen et al. 2008b; Dobruchowska et al. 2012). Besides glucose, the starting substrate maltose and panose [α-d-Glc*p*-(1→6)-α-d-Glc*p*-(1→4)-d-Glc*p*], four compounds could be identified, i.e., α-d-Glc*p*-(1→4)-α-d-Glc*p*-(1→4)-d-Glc*p* (maltotriose), α-d-Glc*p*-(1→6)-α-d-Glc*p*-(1→6)-α-d-Glc*p*-(1→4)-d-Glc*p*, α-d-Glc*p*-(1→6)-α-d-Glc*p*-(1→6)-α-D-Glc*p*-(1→6)-α-d-Glc*p*-(1→4)-d-Glc*p*, and α-d-Glc*p*-(1→6)-α-d-Glc*p*-(1→6)-α-d-Glc*p*-(1→6)-α-d-Glc*p*-(1→6)-α-d-Glc*p*-(1→4)-d-Glc*p* (Fig. 4). For ^1^H NMR spectra, see (Suppl. Info. Fig. S4).

### Enzymatic hydrolysis of the 4,6-αGT-W and 4,6-αGT-ML4 products

**Fig. 5:**
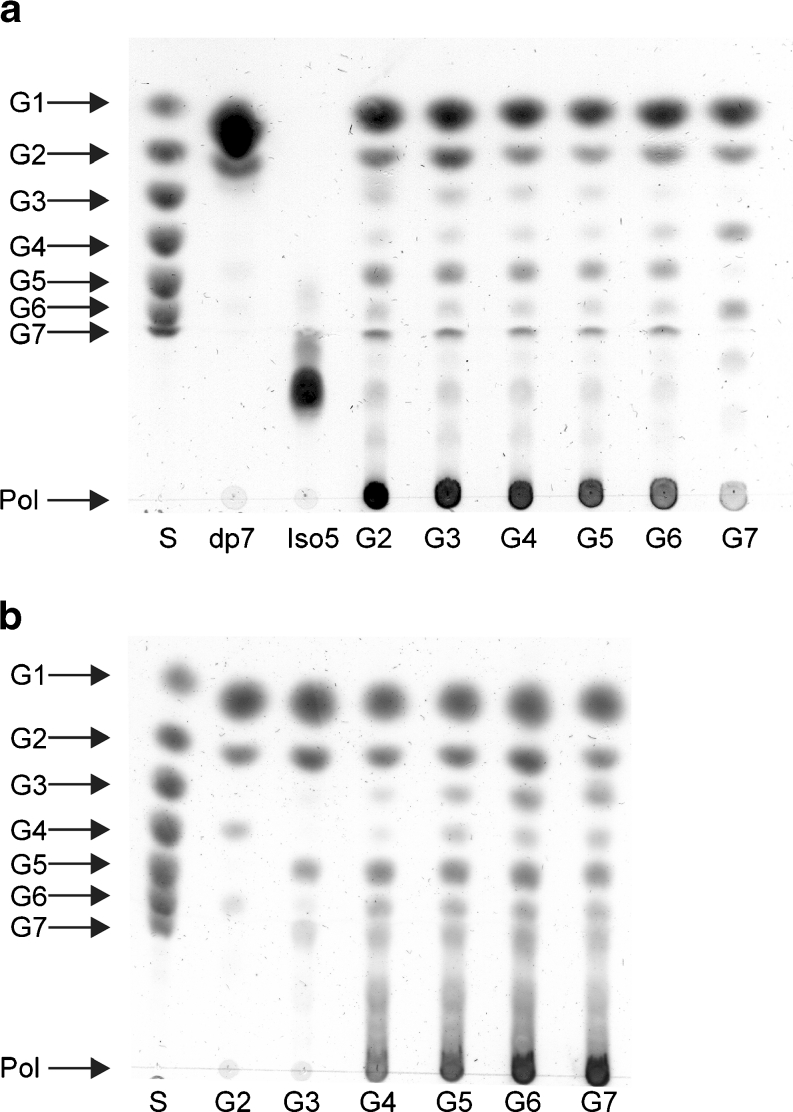
4,6-αGT-W and 4,6-αGT-ML4 generate α-amylase resistant α-glucans. TLC analysis of the product mixtures from maltooligosaccharide (G2-G7) incubations with 4,6-αGT-W (**a**) and 4,6-αGT-ML4 (**b**) (see Fig. 2), after treatment with a high-dose of pig pancreatic α-amylase. *S* standard: glucose (G1) to maltoheptaose (G7); *Pol* polymer. *Lanes 2–7* are the product mixtures from G2 to G7, generated by the sequential 4,6-αGT/α-amylase incubations. The *upper panel* shows as controls isomaltopentaose (Iso5; not degraded by α-amylase) and maltoheptaose (dp7; degraded to maltose and glucose by α-amylase). Note that Iso5 is not entirely pure

To investigate to what extent the α-glucan product mixtures made from maltose up to maltoheptaose using 4,6-αGT-W and 4,6-αGT-ML4 were resistant to α-amylase digestion, they were incubated with a high dose of pig pancreatic α-amylase. Subsequent TLC analysis (Fig. 5) revealed small oligosaccharides but also larger oligosaccharides and polymeric material. These data thus shows that both 4,6-αGTs synthesize α-glucans partly resistant to the endo-α1→4-hydrolase action of α-amylase. The product mixtures are, however, completely degraded to glucose by amyloglucosidase (data not shown), demonstrating that the products are composed of α1→4 and α1→6 glycosidic bonds only, which is in agreement with the ^1^H NMR analysis.

4,6-αGT-W mainly synthesizes α1→6 glycosidic linkages, but also occasionally α1→4 glycosidic bonds, as maltotriose is one of the products made from maltose (Fig. 4). To explore whether 4,6-αGT-W also elongates via an α1→4 glycosidic bond onto oligosaccharides of which the non-reducing glucose moiety is attached via an α1→6 linkage, thereby creating -α-d-Glc*p*-(1→4)-α-d-Glc*p*-(1→6)- elements, the product mixtures obtained from maltose up to maltoheptaose were treated with pullulanase type M1. This enzyme hydrolyzes α1→6 glycosidic linkages in (-)α-d-Glc*p*-(1→4)-α-d-Glc*p*-(1→6)- sequences only. Comparison of the HPAEC elution profiles before and after pullulanase treatment showed only a few differences in the larger oligosaccharide and polymeric fractions (Suppl. Info. Fig. S5), demonstrating that 4,6-αGT-W occasionally synthesizes such a sequence.

## Discussion

It was generally accepted that all GH70 enzymes synthesize α-glucans from sucrose, until we recently demonstrated that *L. reuteri* 121 encodes a GH70 enzyme (called 4,6-αGT-B) that uses (1→4)-α-glucans, but not sucrose, as substrate (Kralj et al. 2011; Dobruchowska et al. 2012). Even though it is known that some GH70 enzymes possess a low disproportionating activity with α-glucans in addition to their main activity (Binder et al. 1983), it was surprising to find a GH70 enzyme to be so effective in utilizing (1→4)-α-glucans as glucose donor because the glycosidic linkage of sucrose is labile compared with the α1→4 glycosidic bond. Phylogenetic tree analysis of the GH70 protein sequences revealed that 4,6-αGT-B clusters with a few hypothetical proteins (Kralj et al. 2011) (Suppl. Info. Fig. S1). Here, we show that the hypothetical GH70 proteins of *L. reuteri* strains DSM 20016 and ML1 encode enzymes that convert maltooligosaccharides [(1→4)-α-d-glucooligosaccharides] into linear α-glucans (in fact isomalto/maltooligosaccharides) with about 50% α1→6 glycosidic bonds. It thus appears that 4,6-αGT activity is a common activity within the GH70 family.

**Fig. 6:**
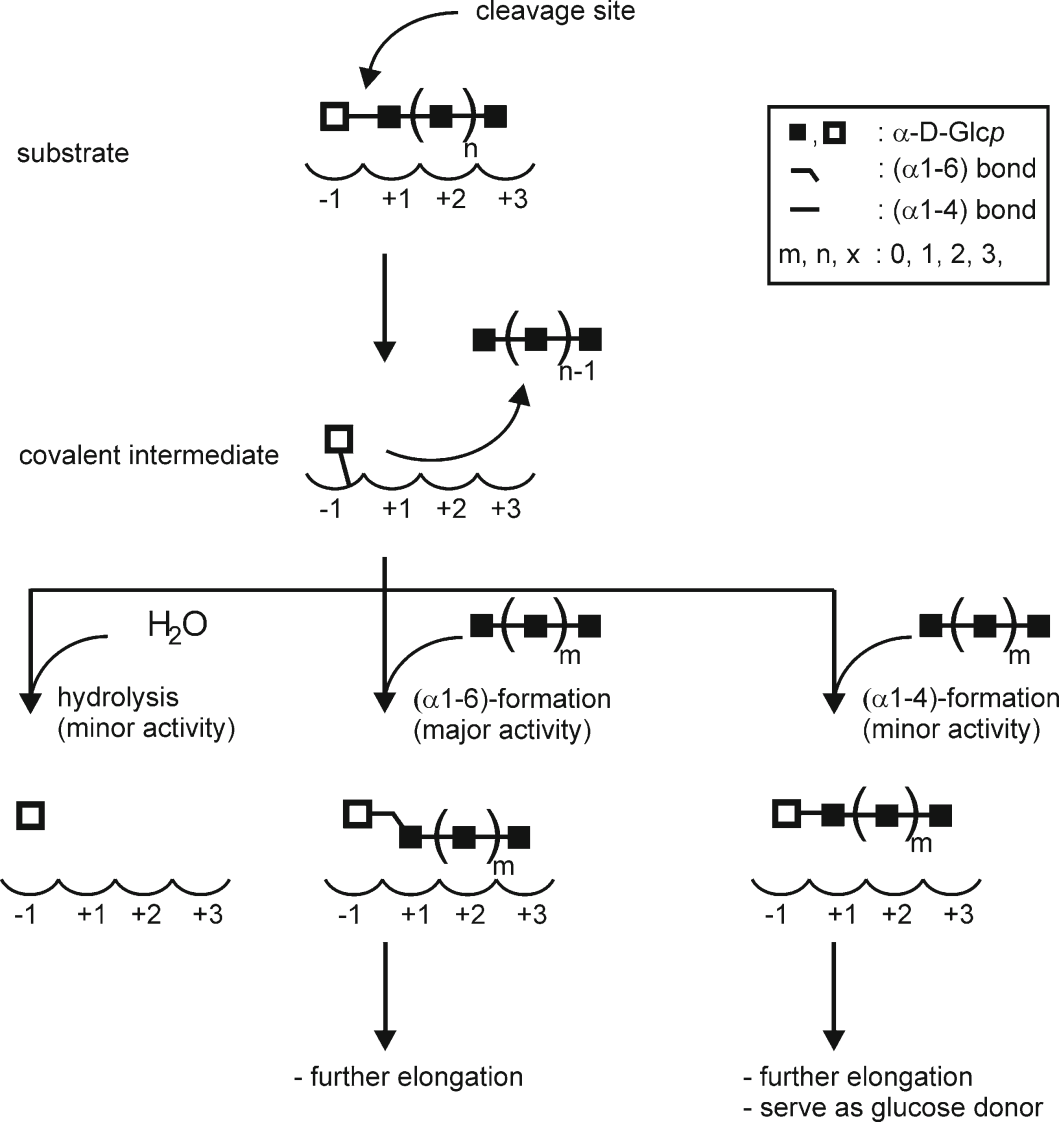
Schematic diagram of the reactions catalyzed by 4,6-αGT enzymes. The *open squares* indicate the glucose moiety transferred by the enzyme. The sugar binding subsite nomenclature is according to Davies et al. (1997), in which the glycosidic bond is cleaved between donor subsite −1 and acceptor subsite +1

The 4,6-αGT products are synthesized by cleaving off the non-reducing glucose moiety of a (1→4)-α-glucan and transferring the glucose moiety to the HO-6 function of the non-reducing end of an acceptor α-glucan chain [a (1→4)-α-glucan or a (1→4)-α-glucan already elongated with a number of (α1→6)-linked glucose residues] (Fig. 6). The enzymes possess, in addition, minor hydrolytic and α1→4 transglycosylase activities. All three disproportionating GH70 4,6-αGT enzymes characterized so far synthesize α1→6 glycosidic bonds and occasionally an α1→4 glycosidic bond. In contrast, the common GH70 GTF enzymes synthesize all four types of α-glycosidic linkages possible. It appears unlikely that this is an intrinsic property of the non-sucrose utilizing type of GH70 enzymes. Therefore in the future α1→2 and α1→3 synthesizing non-sucrose utilizing type of GH70 enzymes may be found encoded by the ever increasing number of bacterial genome sequences, unless of course such synthetic capacities are not beneficial for the bacteria.

### The distinct reaction specificities of 4,6-αGTs and GTFs

Currently it is puzzling why 4,6-αGTs use maltooligosaccharides, but not sucrose, as glucose donor. Because GTFs cleave the glycosidic linkage between the glucosyl and fructosyl moieties of sucrose, and 4,6-αGTs the α1→4 glycosidic linkage between two glucosyl moieties, structural differences are expected at substrate binding subsite +1, which holds either a glucosyl or fructosyl unit (see Fig. 6 for subsite nomenclature). Whether the differences at subsite +1 (Table 1) are the only requirements to interchange the reaction specificities of GH70 enzymes or that additional mutations more remote from the catalytic center are required currently is unknown. In addition, acceptor subsite +2 has a few different amino acids (Table 1), which might contribute to the 4,6-αGT reaction specificity. For GTFs, it is known that amino acid substitutions at subsite +1 (in position 1140) and subsite +2 (in positions 1137 and 1141) drastically alter the ratio of α-glycosidic linkages in the α-glucan polymers made by the enzymes (Shimamura et al. 1994; van Leeuwen et al. 2009). Further mutational analysis of the 4,6-αGT acceptor subsites thus may allow elucidation and modification of their glycosidic linkage specificity.

Another remarkable difference is that 4,6-αGTs synthesize linear α-glucans only, whereas GTFs often produce branched polymers. Since linear α-glucans take up less space than branched ones, 4,6-αGTs may possess a narrower acceptor binding cleft. In this regard, it is noteworthy that the loop connecting domains IV and B (residues 932-943 in GTF-180) is eight residues longer in 4,6-αGTs compared to GTFs (Fig. 1). The crystal structures of GTF-180 and GTF-SI (Vujičić-Žagar et al. 2010; Ito et al. 2011) show that part of this loop delineates the active site; a longer loop at this position thus may result in a more restricted binding cleft in 4,6-αGTs. To further study this hypothesis, 3D structural information is needed, and currently, we are trying to obtain crystal structures of 4,6-αGT proteins.

### Differences and similarities with other α-glucan acting enzymes

Furthermore, enzymes such as 4-α-glucanotransferase, cyclodextrin glucanotransferase, branching enzyme, and dextrin dextrinases disproportionate (1→4)-α-glucans (MacGregor et al. 2001; Cantarel et al. 2009; Zona et al. 2004; Palomo et al. 2011; Kelly et al. 2009a; Naessens et al. 2005). However, these GH13, 57, and 77 enzymes synthesize different products than 4,6-αGTs. Note that the sequences of dextrin dextrinases are unknown, as the genes have not been identified yet. Cyclodextrin glucanotransferases make cyclic (1→4)-α-glucans (e.g., cyclodextrins), 4-α-glucanotransferases cleave and synthesize α1→4 glycosidic bonds thereby altering the (1→4)-α-glucan chain distribution, and branching enzymes cleave α1→4 glycosidic bonds and transfer the cleaved-off chain to a HO-6 group within a (1→4)-α-glucan, yielding an α1→6 branch. These enzymes cleave internal α1→4 glycosidic linkages (i.e., endo-acting), whereas 4,6-αGTs are exo-acting enzymes that cleave off the non-reducing glucose unit from an (1→4)-α-glucan chain.

Moreover, cyclodextrin glucanotransferases and 4-α-glucanotransferases synthesize α1→4 glycosidic bonds, whereas 4,6-αGTs make α1→6 bonds. Although branching enzymes also form α1→6 bonds, they create α1→6 bonds in the middle of α-glucan chains, resulting in branch points, whereas 4,6-αGTs attach the glucose unit via an α1→6 glycosidic linkage to the non-reducing end of an α-glucan chain yielding linear products.

The reaction specificity of dextran dextrinases comes closest to that of 4,6-αGTs, both transferring single glucose moieties from the non-reducing end of maltooligosaccharides to synthesize isomalto/maltooligosaccharides (Yamamoto et al. 1993a; Kralj et al. 2011). The crucial difference between the two types of enzymes is that dextrin dextrinases form branched carbohydrates (Yamamoto et al. 1993b; Wang et al. 2011; Sims et al. 2001; Tsusaki et al. 2009). A second difference is that dextrin dextrinases disproportionate isomaltooligosaccharides (Yamamoto et al. 1993a), whereas isomaltooligosaccharides are not a substrate of 4,6-αGTs. This is relevant as this means that dextran dextrinases can disproportionate their own reaction products, whereas 4,6-αGTs cannot. A key question that remains to be answered is whether dextran dextrinases are evolutionary related to the GH70 enzymes. Based on their activity they combine the disproportionation activity of 4,6-αGTs and the branching activity of GH70 glucansucrases. The answer to this question requires the identification of the dextran dextrinase (gene) sequence.

A further difference is that GH70 enzymes, including 4,6-αGTs, are more hydrolytic than the GH13 and GH77 transglycosylases. For example, the *k*_cat(transglycosylation)_/*k*_cat(hydrolysis)_ ratio of the GH13 *Neisseria polysaccharea* amylosucrase is 20 (Albenne et al. 2004), that of GH13 *Bacillus circulans* CGTase is 90 (van der Veen et al. 2001), GH13 branching enzymes have ratios of >>1,000, and the GH77 4-α-glucanotransferases of *Thermus thermophilus* and *T. brockianus* have ratios of 5,000 (Kaper et al. 2007; Jung et al. 2011). GH70 GTFs, in contrast, are predominantly hydrolytic in the early phase of sucrose conversion, and they become transglycosylases when better acceptor substrates become available as result of the transglycosylation activity or when good acceptor substrates are added at the start of a reaction (Kralj et al. 2004b). Here, we find that also 4,6-αGT-W has about 50% hydrolytic activity at the start of a reaction with low concentrations of linear (1→4)-α-d-glucooligosaccharides and becomes a better transglycosylase once products appear that again function as acceptor substrate. The acceptor subsites of GH70 enzymes seem thus to be optimized for recognizing and utilizing the products made by the enzyme as acceptor substrates in a polymerization reaction.

### Applications and future perspectives of 4,6-αGTs

4,6-αGTs are an exciting type of novel enzymes that convert (1→4)-α-glucan substrates into linear α-glucan products with a high percentage of α1→6 glycosidic linkages (i.e., isomaltooligosaccharides built on maltooligosaccharides). These enzymes are potentially very useful for the synthesis of carbohydrate displaying compounds. Indeed, GH13 and GH70 enzymes are being used for the glycosylation of a variety of compounds (Desmet and Soetaert 2011; Leemhuis et al. 2010; Homann and Seibel 2009). The fact that 4,6-αGTs synthesizes mainly α1→6 glycosidic bonds ensures that the products are water-soluble, which is beneficial for increasing the solubility of poorly water-soluble compounds such as drug molecules.

Because 4,6-αGTs generate α-glucans with a high degree of α1→6 bonds, their products are water-soluble, which is advantageous for applications such as energy and soft drinks. The α1→6 linkages, in addition, make the products resistant to α-amylase digestion and the products as such are expected to pass the human stomach and small intestine and enter the colon were they can serve as carbon source for health promoting bacteria. Thus, 4,6-αGTs are expected to convert readily degradable maltooligosaccharides into a novel type of “resistant” α-glucans and are thus potentially of great interest to the food industry (Dijkhuizen et al. 2011).

## Electronic supplementary material

Below is the link to the electronic supplementary material.

ESM 1 (DOC 3.36 MB)

## References

[CR1] Albenne C, Skov LK, Mirza O, Gajhede M, Feller G, D'Amico S, André G, Potocki-Véronèse G, van der Veen BA, Monsan P, Remaud-Siméon M (2004). Molecular basis of the amylose-like polymer formation catalyzed by *Neisseria polysaccharea* amylosucrase. J Biol Chem.

[CR2] Bendtsen JD, Nielsen H, von Heijne G, Brunak S (2004). Improved prediction of signal peptides: SignalP 3.0. J Mol Biol.

[CR3] Binder TP, Côté GL, Robyt JF (1983). Disproportionation reactions catalyzed by *Leuconostoc* and *Streptococcus* glucansucrases. Carbohydr Res.

[CR4] Brison Y, Pijning T, Malbert Y, Fabre E, Mourey L, Morel S, Potocki-Véronèse G, Monsan P, Tranier S, Remaud-Siméon M, Dijkstra BW (2012) Functional and structural characterization of an α-(1→2) branching sucrase derived from DSR-E glucansucrase. J Biol Chem. doi:10.1074/jbc.M111.305078 10.1074/jbc.M111.305078 PMC3318707 22262856

[CR5] Cantarel BL, Coutinho PM, Rancurel C, Bernard T, Lombard V, Henrissat B (2009). The Carbohydrate-Active EnZymes database (CAZy): an expert resource for glycogenomics. Nucleic Acids Res.

[CR6] Ciucanu I, Kerek F (1984). A simple and rapid method for the permethylation of carbohydrates. Carbohydr Res.

[CR7] Côté GL, Robyt JF (1982). Isolation and partial characterization of an extracellular glucansucrase from *Leuconostoc mesenteroides* NRRL B-1355 that synthesizes an alternating (1→6), (1→3)-α-D-glucan. Carbohydr Res.

[CR8] Davies GJ, Wilson KS, Henrissat B (1997). Nomenclature for sugar-binding subsites in glycosyl hydrolases. Biochem J.

[CR9] Desmet T, Soetaert W (2011). Enzymatic glycosyl transfer: mechanisms and applications. Biocatal Biotransform.

[CR10] Dijkhuizen L, van der Maarel MJEC, Kamerling JP, Leemhuis H, Kralj S, Dobruchowska JM (2010) Gluco-oligosaccharides comprising (α1-4) and (α1-6) glycosidic bonds, use thereof, and methods for providing them. WO/2010/128859

[CR11] Dobruchowska JM, Gerwig GJ, Kralj S, Grijpstra P, Leemhuis H, Dijkhuizen L, Kamerling JP (2012) Structural characterization of linear isomalto/malto-oligomer products synthesized by the novel GTFB 4,6-α-glucanotransferase enzyme from *Lactobacillus reuteri* 121. Glycobiology. doi:10.1093/glycob/cwr167 10.1093/glycob/cwr167 22138321

[CR12] Domań-Pytka M, Bardowski J (2004). Pullulan degrading enzymes of bacterial origin. Crit Rev Microbiol.

[CR13] Fabre E, Bozonnet S, Arcache A, Willemot R-M, Vignon M, Monsan P, Remaud-Siméon M (2005). Role of the two catalytic domains of DSR-E dextransucrase and their involvement in the formation of highly α-1,2 branched dextran. J Bacteriol.

[CR14] Goujon M, McWilliam H, Li W, Valentin F, Squizzato S, Paern J, Lopez R (2010). A new bioinformatics analysis tools framework at EMBL-EBI. Nucleic Acids Res.

[CR15] Hellmuth H, Wittrock S, Kralj S, Dijkhuizen L, Hofer B, Seibel J (2008). Engineering the glucansucrase GTFR enzyme reaction and glycosidic bond specificity: toward tailor-made polymer and oligosaccharide products. Biochemistry.

[CR16] Homann A, Seibel J (2009). Chemo-enzymatic synthesis and functional analysis of natural and modified glycostructures. Nat Prod Rep.

[CR17] Irague R, Massou S, Moulis C, Saurel O, Milon A, Monsan P, Remaud-Siméon M, Portais J-C, Potocki-Véronèse G (2011). NMR-based structural glycomics for high-throughput screening of carbohydrate-active enzyme specificity. Anal Chem.

[CR18] Ito K, Ito S, Shimamura T, Weyand S, Kawarasaki Y, Misaka T, Abe K, Kobayashi T, Cameron AD, Iwata S (2011). Crystal structure of glucansucrase from the dental caries pathogen *Streptococcus mutans*. J Mol Biol.

[CR19] Jung J-H, Jung T-Y, Seo D-H, Yoon S-M, Choi H-C, Park BC, Park C-S, Woo E-J (2011). Structural and functional analysis of substrate recognition by the 250s loop in amylomaltase from *Thermus brockianus*. Proteins.

[CR20] Kaditzky SB, Behr J, Stocker A, Kaden P, Ganzle MG, Vogel RF (2008). Influence of pH on the formation of glucan by *Lactobacillus reuteri* TMW 1.106 exerting a protective function against extreme pH values. Food Biotechnol.

[CR21] Kaper T, Leemhuis H, Uitdehaag JCM, van der Veen BA, Dijkstra BW, van der Maarel MJEC, Dijkhuizen L (2007). Identification of acceptor substrate binding subsites +2 and +3 in the amylomaltase from *Thermus thermophilus* HB8. Biochemistry.

[CR22] Kelly RM, Dijkhuizen L, Leemhuis H (2009). Starch and α-glucan acting enzymes, modulating their properties by directed evolution. J Biotechnol.

[CR23] Kelly RM, Dijkhuizen L, Leemhuis H (2009). The evolution of cyclodextrin glucanotransferase product specificity. Appl Microbiol Biotechnol.

[CR24] Kralj S, van Geel-Schutten GH, Rahaoui H, Leer RJ, Faber EJ, van der Maarel MJEC, Dijkhuizen L (2002). Molecular characterization of a novel glucosyltransferase from *Lactobacillus reuteri* strain 121 synthesizing a unique, highly branched glucan with α-(1→4) and α-(1→6) glucosidic bonds. Appl Environ Microbiol.

[CR25] Kralj S, van Geel-Schutten GH, Dondorff MMG, Kirsanovs S, van der Maarel MJEC, Dijkhuizen L (2004). Glucan synthesis in the genus *Lactobacillus*: isolation and characterization of glucansucrase genes, enzymes and glucan products from six different strains. Microbiology.

[CR26] Kralj S, van Geel-Schutten GH, van der Maarel MJEC, Dijkhuizen L (2004). Biochemical and molecular characterization of *Lactobacillus reuteri* 121 reuteransucrase. Microbiology.

[CR27] Kralj S, Grijpstra P, van Leeuwen SS, Leemhuis H, Dobruchowska JM, van der Kaaij RM, Malik A, Oetari A, Kamerling JP, Dijkhuizen L (2011). 4,6-α-Glucanotransferase, a novel enzyme that structurally and functionally provides an evolutionary link between glycoside hydrolase enzyme families 13 and 70. Appl Environ Microbiol.

[CR28] Larkin MA, Blackshields G, Brown NP, Chenna R, McGettigan PA, McWilliam H, Valentin F, Wallace IM, Wilm A, Lopez R, Thompson JD, Gibson TJ, Higgins DG (2007). Clustal W and Clustal X version 2.0. Bioinformatics.

[CR29] Leemhuis H, Kelly RM, Dijkhuizen L (2010). Engineering of cyclodextrin glucanotransferases and the impact for biotechnological applications. Appl Microbiol Biotechnol.

[CR30] MacGregor EA, Janecek S, Svensson B (2001). Relationship of sequence and structure to specificity in the α-amylase family of enzymes. Biochim Biophys Acta.

[CR31] Monchois V, Willemot R-M, Monsan P (1999). Glucansucrases: mechanism of action and structure-function relationships. FEMS Microbiol Rev.

[CR32] Morita H, Toh H, Fukuda S, Horikawa H, Oshima K, Suzuki T, Murakami M, Hisamatsu S, Kato Y, Takizawa T, Fukuoka H, Yoshimura T, Itoh K, O'Sullivan DJ, McKay LL, Ohno H, Kikuchi J, Masaoka T, Hattori M (2008). Comparative genome analysis of *Lactobacillus reuteri* and *Lactobacillus fermentum* reveal a genomic island for reuterin and cobalamin production. DNA Res.

[CR33] Moulis C, Joucla G, Harrison D, Fabre E, Potocki-Veronese G, Monsan P, Remaud-Simeon M (2006). Understanding the polymerization mechanism of glycoside-hydrolase family 70 glucansucrases. J Biol Chem.

[CR34] Naessens M, Cerdobbel A, Soetaert W, Vandamme EJ (2005). Dextran dextrinase and dextran of *Gluconobacter oxydans*. J Ind Microbiol Biotechnol.

[CR35] Palomo M, Pijning T, Booiman T, Dobruchowska JM, van der Vlist J, Kralj S, Planas A, Loos K, Kamerling JP, Dijkstra BW, van der Maarel MJEC, Dijkhuizen L, Leemhuis H (2011). *Thermus thermophilus* glycoside hydrolase family 57 branching enzyme: crystal structure, mechanism of action, and products formed. J Biol Chem.

[CR36] Potocki de Montalk G, Remaud-Siméon M, Willemot R-M, Sarçabal P, Planchot V, Monsan P (2000). Amylosucrase from *Neisseria polysaccharea*: novel catalytic properties. FEBS Lett.

[CR37] Schwab C, Walter J, Tannock GW, Vogel RF, Gänzle MG (2007). Sucrose utilization and impact of sucrose on glycosyltransferase expression in *Lactobacillus reuteri*. Syst Appl Microbiol.

[CR38] Shimamura A, Nakano YJ, Mukasa H, Kuramitsu HK (1994). Identification of amino acid residues in *Streptococcus mutans* glucosyltransferases influencing the structure of the glucan product. J Bacteriol.

[CR39] Sims IM, Thomson A, Hubl U, Larsen NG, Furneaux RH (2001). Characterisation of polysaccharides synthesised by *Gluconobacter oxydans* NCIMB 4943. Carbohydr Polym.

[CR40] Stam MR, Danchin EGJ, Rancurel C, Coutinho PM, Henrissat B (2006). Dividing the large glycoside hydrolase family 13 into subfamilies: towards improved functional annotations of α-amylase-related proteins. Protein Eng Des Sel.

[CR41] Tsusaki K, Watanabe H, Nishimoto T, Yamamoto T, Kubota M, Chaen H, Fukuda S (2009). Structure of a novel highly branched α-glucan enzymatically produced from maltodextrin. Carbohydr Res.

[CR42] van der Veen BA, Leemhuis H, Kralj S, Uitdehaag JCM, Dijkstra BW, Dijkhuizen L (2001). Hydrophobic amino acid residues in the acceptor binding site are main determinants for reaction mechanism and specificity of cyclodextrin-glycosyltransferase. J Biol Chem.

[CR43] van Hijum SAFT, Kralj S, Ozimek LK, Dijkhuizen L, van Geel-Schutten IGH (2006). Structure-function relationships of glucansucrase and fructansucrase enzymes from lactic acid bacteria. Microbiol Mol Biol Rev.

[CR44] van Leeuwen SS, Kralj S, van Geel-Schutten IH, Gerwig GJ, Dijkhuizen L, Kamerling JP (2008). Structural analysis of the α-D-glucan (EPS35-5) produced by the *Lactobacillus reuteri* strain 35-5 glucansucrase GTFA enzyme. Carbohydr Res.

[CR45] van Leeuwen SS, Leeflang BR, Gerwig GJ, Kamerling JP (2008). Development of a ^1^H NMR structural-reporter-group concept for the primary structural characterisation of α-D-glucans. Carbohydr Res.

[CR46] van Leeuwen SS, Kralj S, Eeuwema W, Gerwig GJ, Dijkhuizen L, Kamerling JP (2009). Structural characterization of bioengineered α-D-glucans produced by mutant glucansucrase GTF180 enzymes of *Lactobacillus reuteri* strain 180. Biomacromolecules.

[CR47] Vujičić-Žagar A, Pijning T, Kralj S, López CA, Eeuwema W, Dijkhuizen L, Dijkstra BW (2010). Crystal structure of a 117 kDa glucansucrase fragment provides insight into evolution and product specificity of GH70 enzymes. Proc Natl Acad Sci USA.

[CR48] Walter J, Schwab C, Loach DM, Gänzle MG, Tannock GW (2008). Glucosyltransferase A (GtfA) and inulosucrase (Inu) of *Lactobacillus reuteri* TMW1.106 contribute to cell aggregation, in vitro biofilm formation, and colonization of the mouse gastrointestinal tract. Microbiology.

[CR49] Wang S, Mao X, Wang H, Lin J, Li F, Wei D (2011). Characterization of a novel dextran produced by *Gluconobacter oxydans* DSM 2003. Appl Microbiol Biotechnol.

[CR50] Yamamoto K, Yoshikawa K, Okada S (1993). Detailed action mechanism of dextrin dextranase from *Acetobacter capsulatus* ATCC 11894. Biosci Biotechnol Biochem.

[CR51] Yamamoto K, Yoshikawa K, Okada S (1993). Structure of dextran synthesized by dextrin dextranase from *Acetobacter capsulatus* ATCC 11894. Biosci Biotechnol Biochem.

[CR52] Zona R, Chang-Pi-Hin F, O'Donohue MJ, Janeček S (2004). Bioinformatics of the glycoside hydrolase family 57 and identification of catalytic residues in amylopullulanase from *Thermococcus hydrothermalis*. Eur J Biochem.

