# Supplementary material for: 4,6-α-Glucanotransferase activity occurs more widespread in Lactobacillus strains and constitutes a separate GH70 subfamily
Source: Appl Microbiol Biotechnol. 2012 Feb 25;97(1):181–93. doi: 10.1007/s00253-012-3943-1 (PMC3536977; doi:10.1007/s00253-012-3943-1)
Supplement: Supplementary file 1 — (DOC 3.36 MB) [file 253_2012_3943_MOESM1_ESM.doc]

**Supplemental Information to:**

**4,6-α-Glucanotransferase activity occurs more widespread in**

***Lactobacillus* strains and constitutes a separate GH70 subfamily**

Hans Leemhuis, Willem P. Dijkman, Justyna M. Dobruchowska, Tjaard Pijning, Pieter Grijpstra, Slavko Kralj, Johannis P. Kamerling, Lubbert Dijkhuizen

**Contents page**

**Fig. S1** Phylogenetic tree of GH70 enzymes 2

**Fig. S2** HPAEC elution profiles of reaction mixtures 3

**Fig. S3** Maltotetraose conversion by 4,6-αGT-W in time 4

**Fig. S4** ^1^H NMR spectra of oligosaccharides generated from 5 – 6

maltose by 4,6-αGT-W

**Fig. S5** Pullulanase digestion of products generated from 7

maltotetraose by 4,6-αGT-W

**References**  7


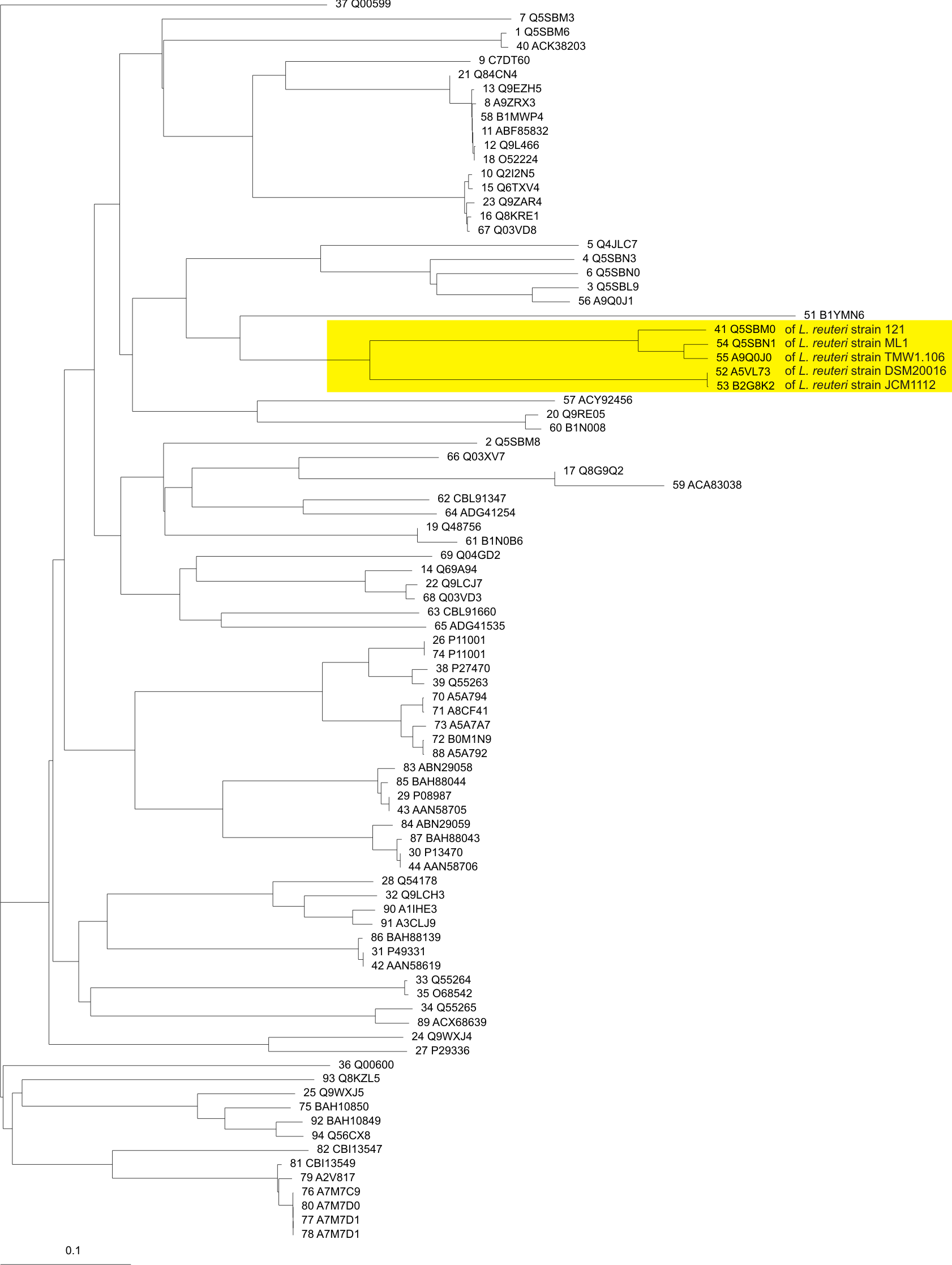


**Fig. S1** Dendrogram of the GH70 protein sequences available via the Carbohydrate-Active Enzymes Database CAZy at [http://www.cazy.org](http://www.cazy.org/) (Cantarel et al., 2009). The (putative) 4,6-αGT sequences form a cluster in the dendrogram and are highlighted with a yellow background. The proteins are indicated with their Uniprot code and for the (putative) 4,6-αGTs also the organisms name is provided.


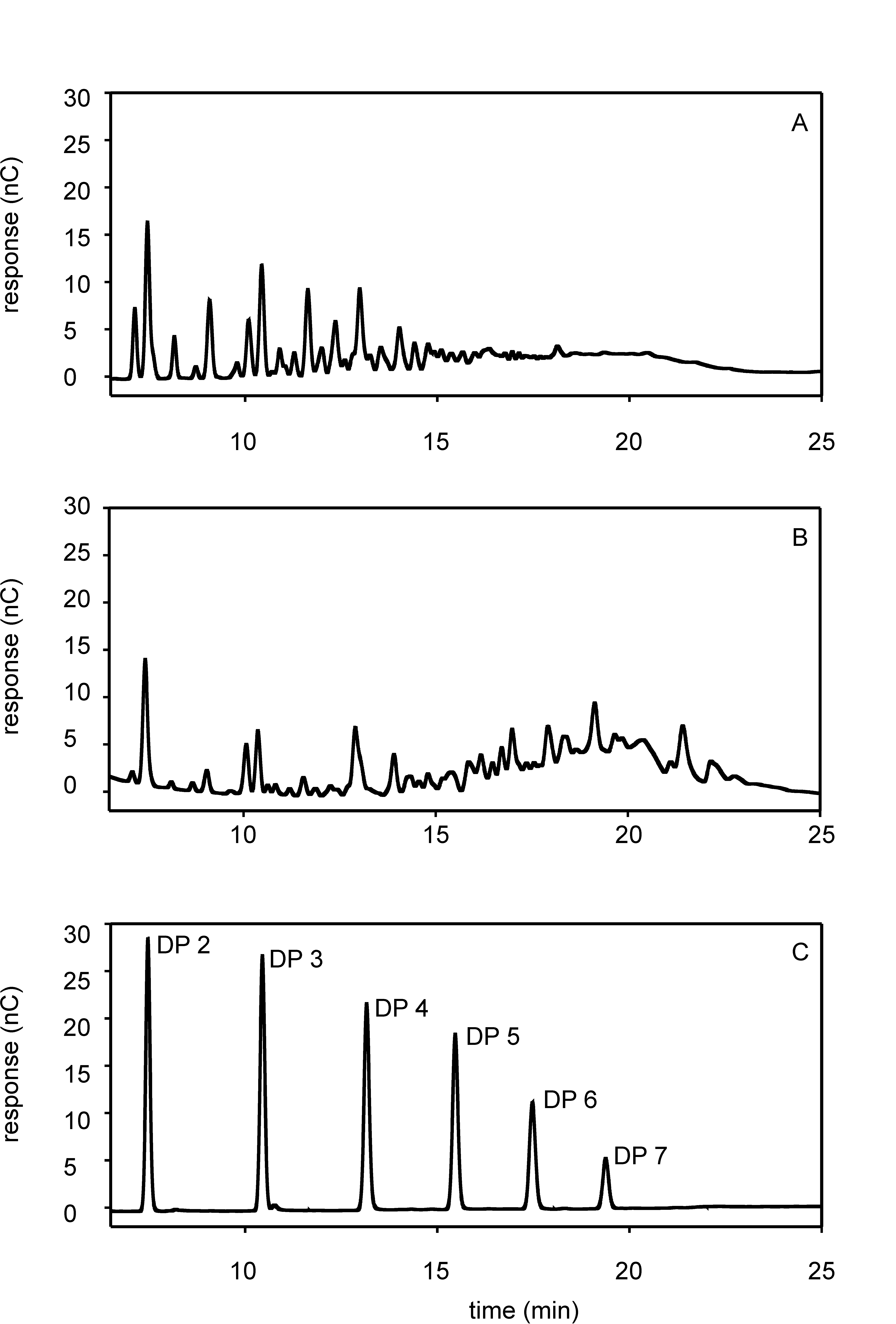


**Fig. S2.** HPAEC elution profiles of reaction mixtures obtained from maltoheptaose following incubation with (A) 4,6-αGT-W and (B) 4,6-αGT-ML4. Panel C shows the elution pattern of a (1→4)-α-D-glucooligosaccharide standard from DP 2 to DP 7.


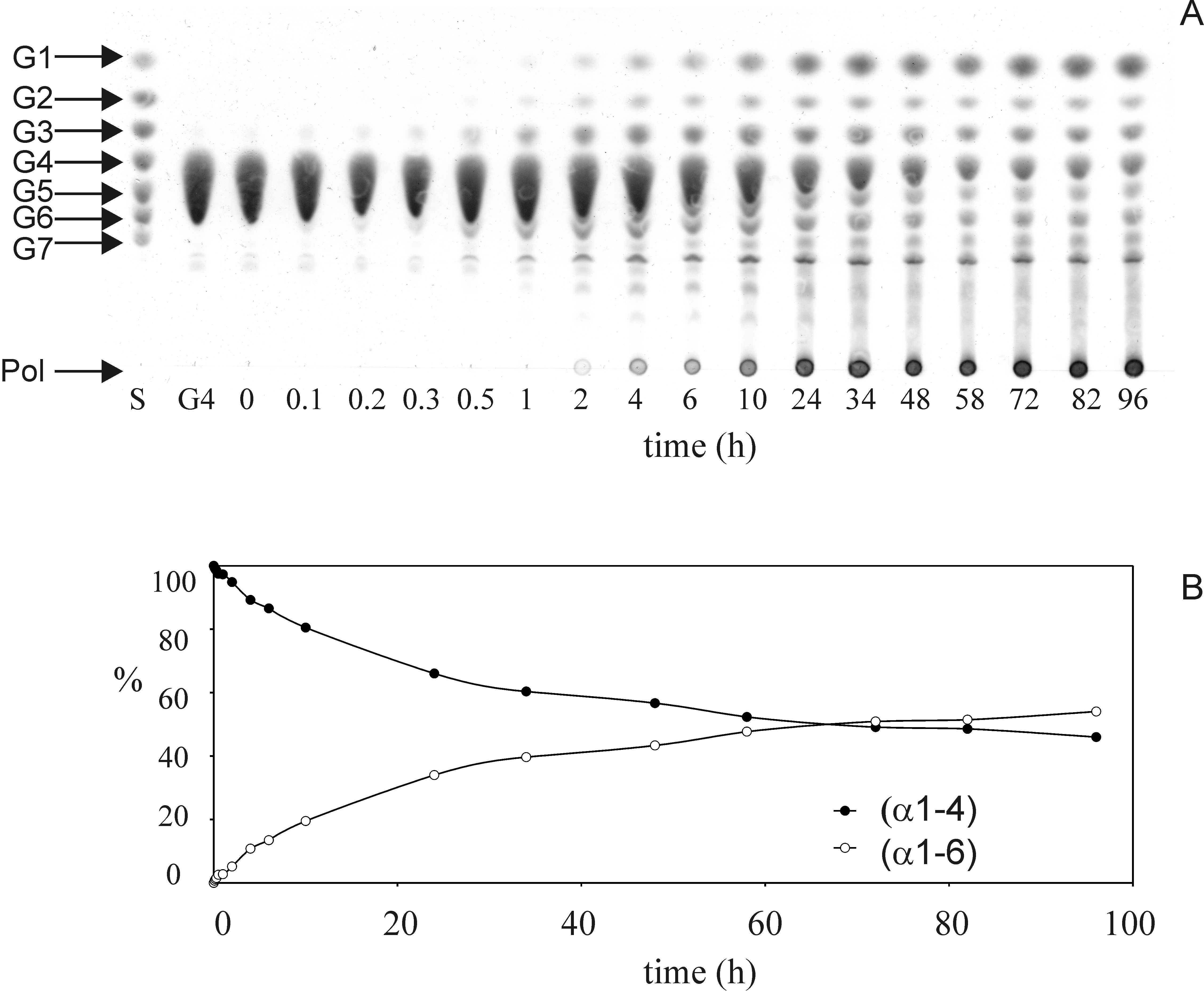


**Fig. S3** Maltotetraose conversion by 4,6-αGT-W in time.

The progress of the reaction is followed in time by (A) TLC analysis and (B) ^1^H NMR analysis (the percentages of the α1→4 and α1→6 glycosidic linkages are indicated with filled and open circles, respectively). Reaction conditions: 90 mg maltotetraose; 13 μg 4,6-αGT-W enzyme; pH 4.7 and 37°C. At the end of the incubation the fraction of α1→6 glycosidic bonds had increased from 0 to 0.57. The enzyme has a low hydrolytic activity as the α- plus β-anomeric signals increased by 15% relative to the total of the H-1 signals that reflect the α1→4 and α1→6 glycosidic bonds (^1^H NMR spectra not shown).

**
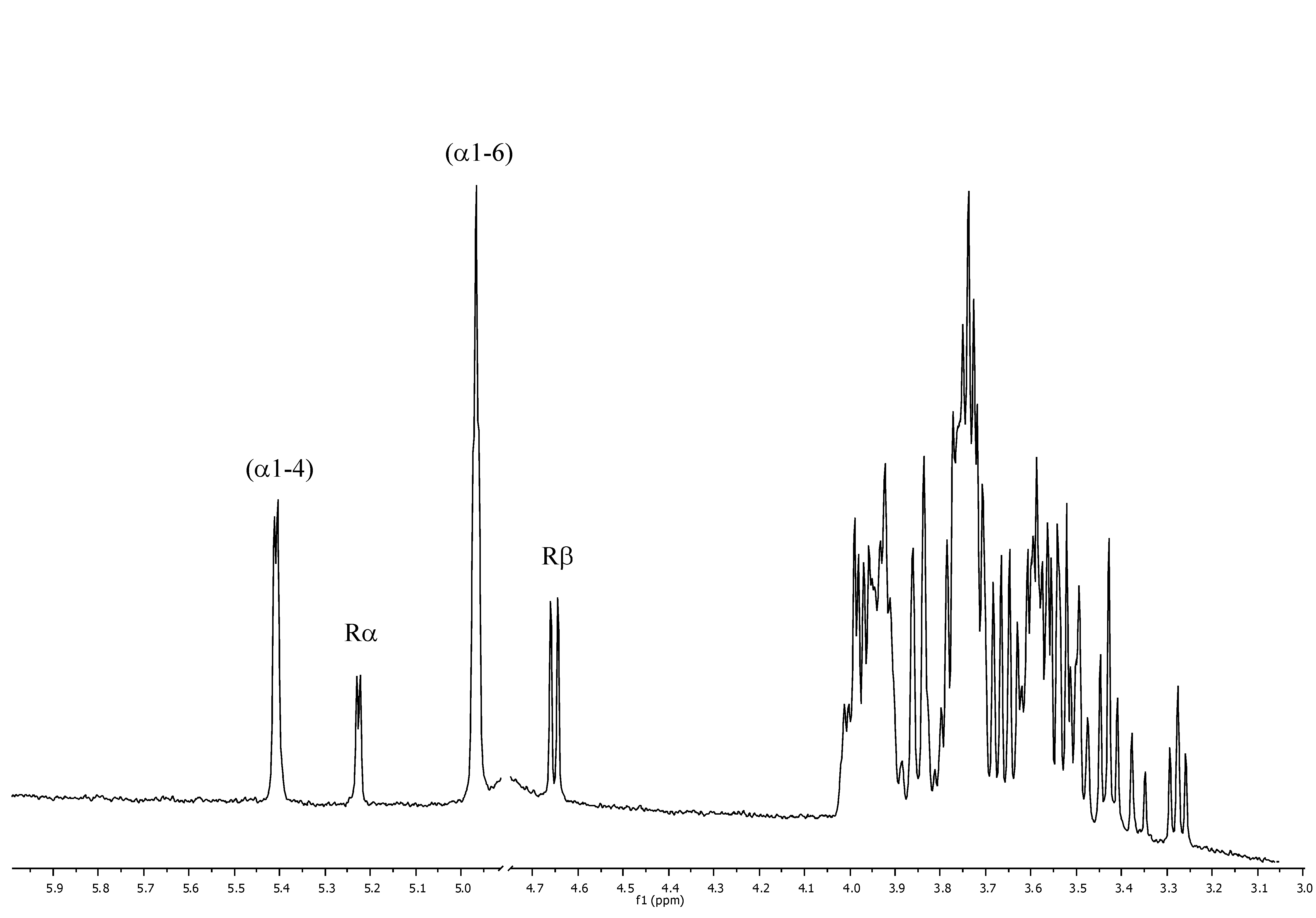
**

**Fig. S4a** ^1^H NMR spectrum of HPAEC peak 1 (see Fig.4 in the manuscript), isolated from the product mixture generated from maltose by incubation with 4,6-αGT-W. The spectrum is identical to the ^1^H NMR spectrum of α-D-Glc*p*-(1→6)-α-D-Glc*p*-(1→6)-α-D-Glc*p*-(1→4)-D-Glc*p* (Dobruchowska et al. 2012).


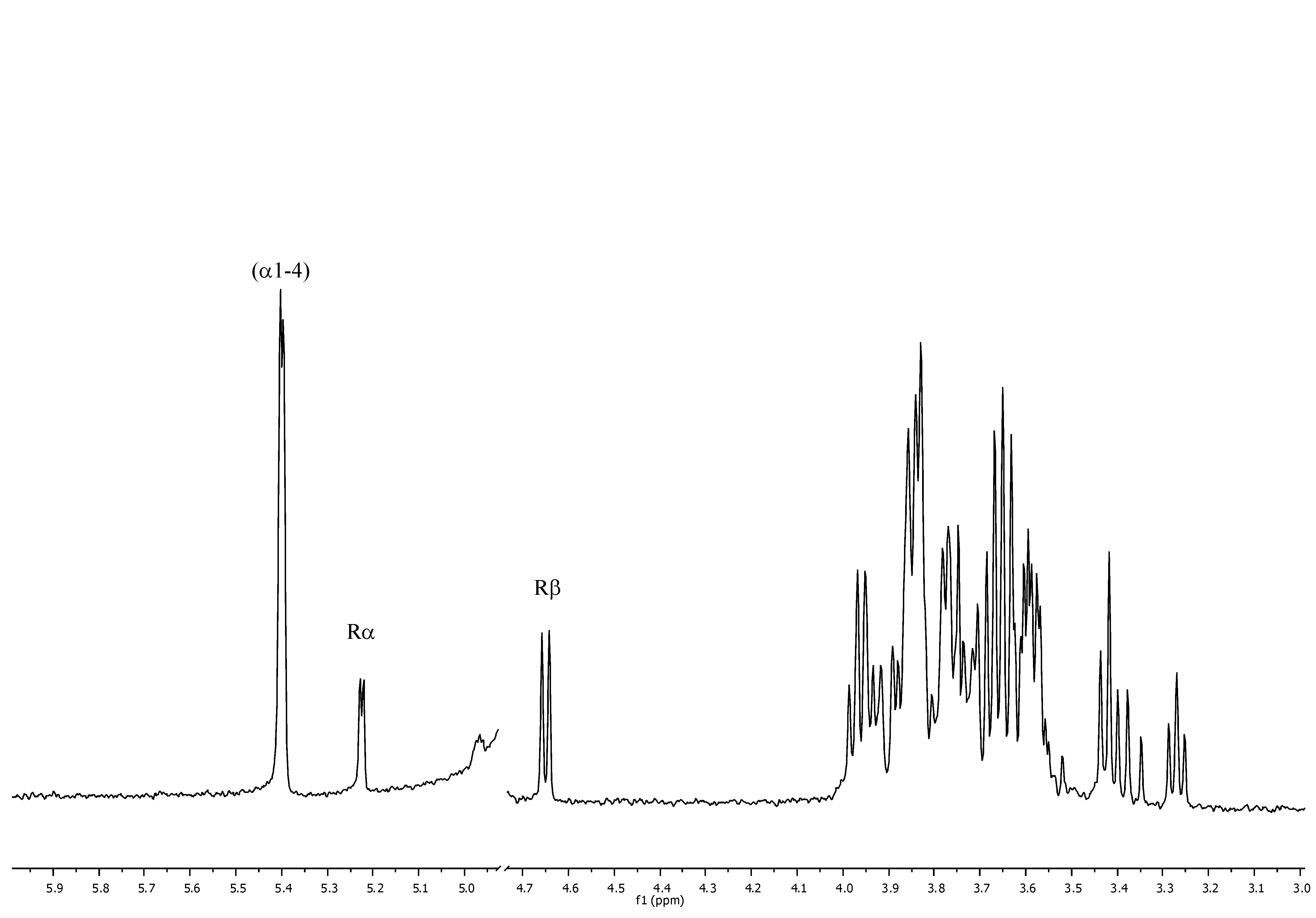


**Fig. S4b** ^1^H NMR spectrum of HPAEC peak 2 (see Fig.4 in the manuscript), isolated from the product mixture generated from maltose by incubation with 4,6-αGT-W. The spectrum is identical to the ^1^H NMR spectrum of α-D-Glc*p*-(1→4)-α-D-Glc*p*-(1→4)-D-Glc*p* (maltotriose) (Dobruchowska et al. 2012).


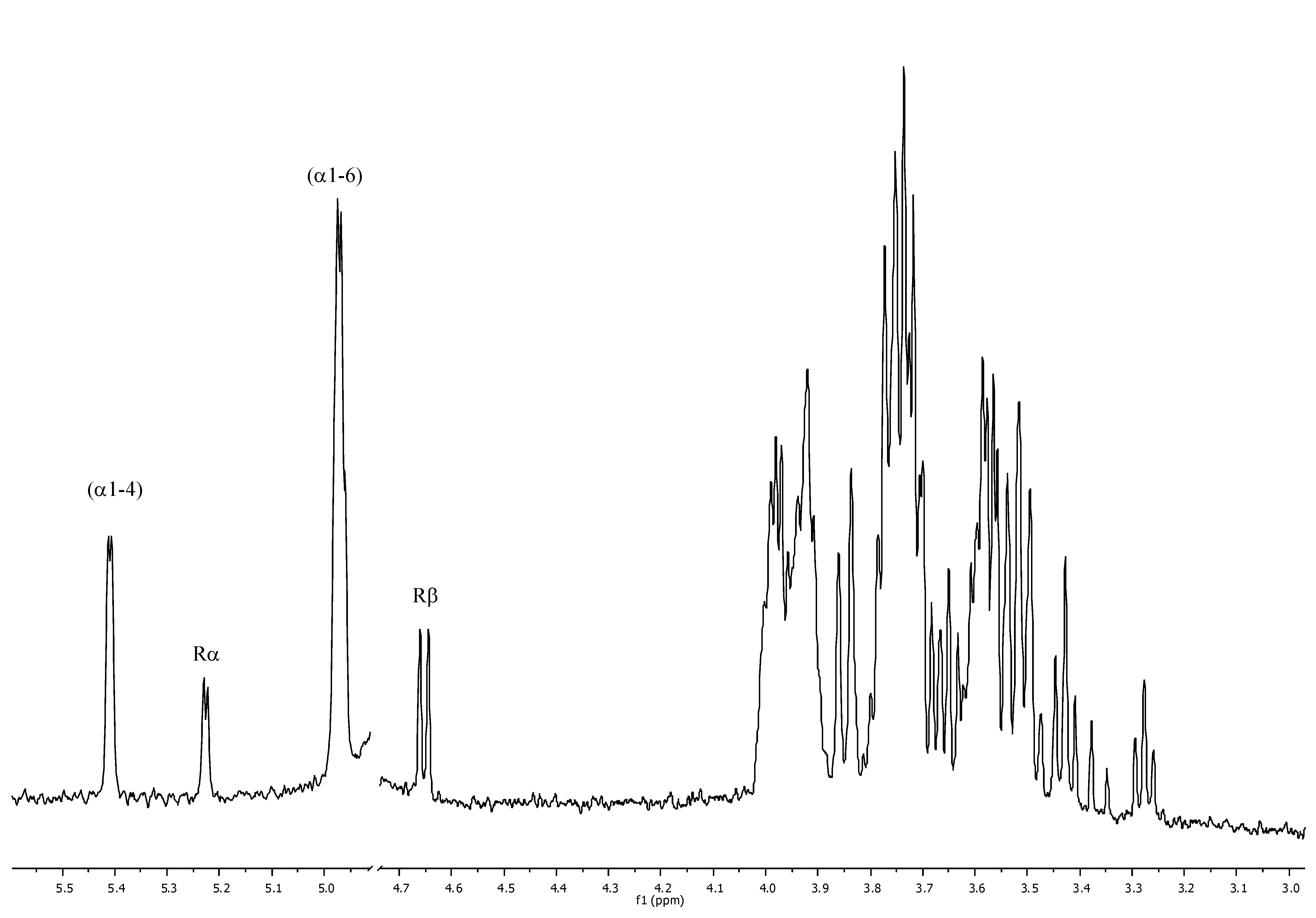


**Fig. S4c** ^1^H NMR spectrum of HPAEC peak 3 (see Fig.4 in the manuscript), isolated from the product mixture generated from maltose by incubation with 4,6-αGT-W. The spectrum is identical to the ^1^H NMR spectrum of α-D-Glc*p*-(1→6)-α-D-Glc*p*-(1→6)-α-D-Glc*p*-(1→6)-α-D-Glc*p*-(1→4)-D-Glc*p* (Dobruchowska et al. 2012).


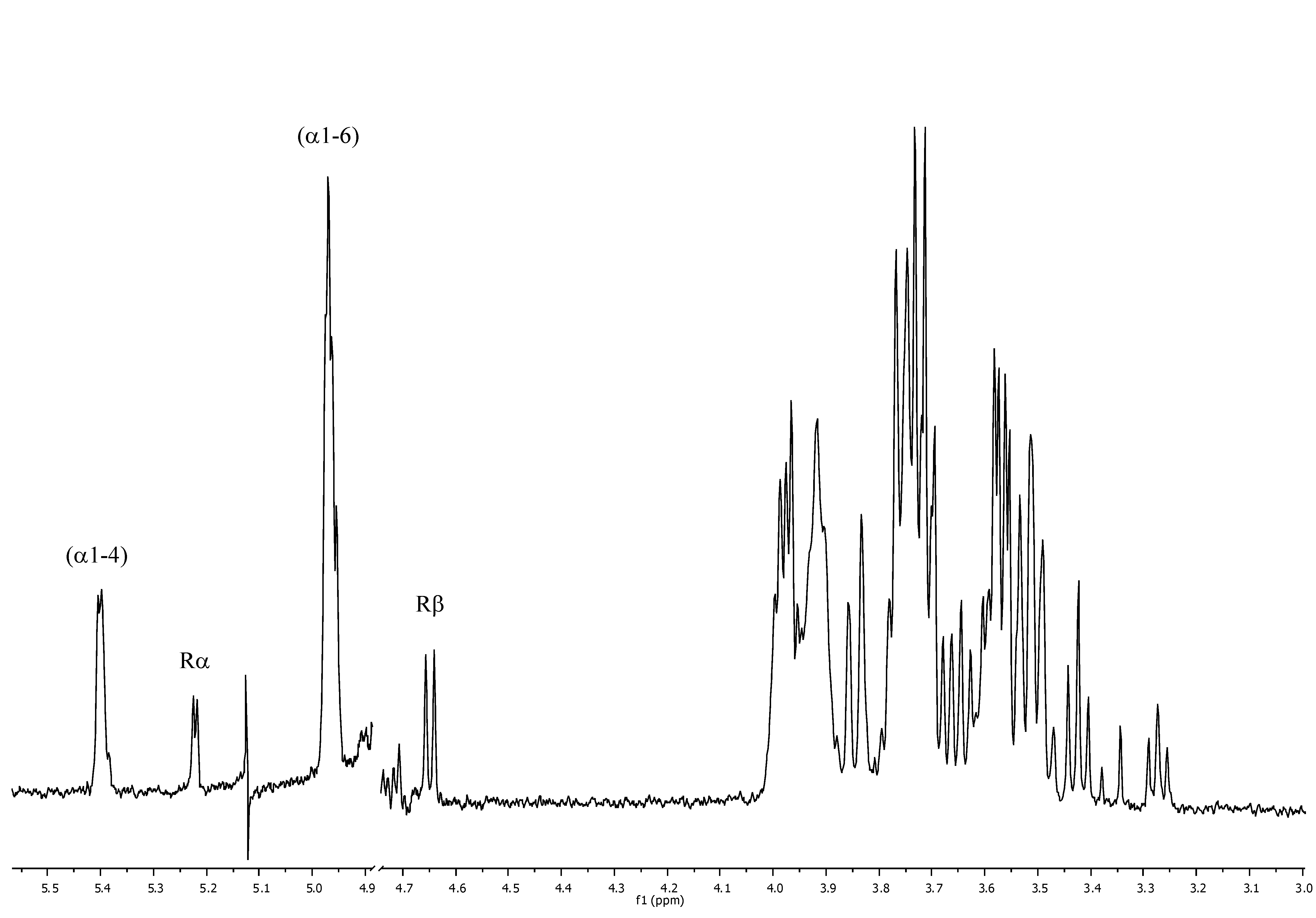


**Fig. S4d** ^1^H NMR spectrum of HPAEC peak 4 (see Fig.4 in the manuscript), isolated from the product mixture generated from maltose by incubation with 4,6-αGT-W. The spectrum is identical to the ^1^H NMR spectrum of α-D-Glc*p*-(1→6)-α-D-Glc*p*-(1→6)-α-D-Glc*p*-(1→6)-α-D-Glc*p*-(1→6)-α-D-Glc*p*-(1→4)-D-Glc*p* (Dobruchowska et al., 2012).


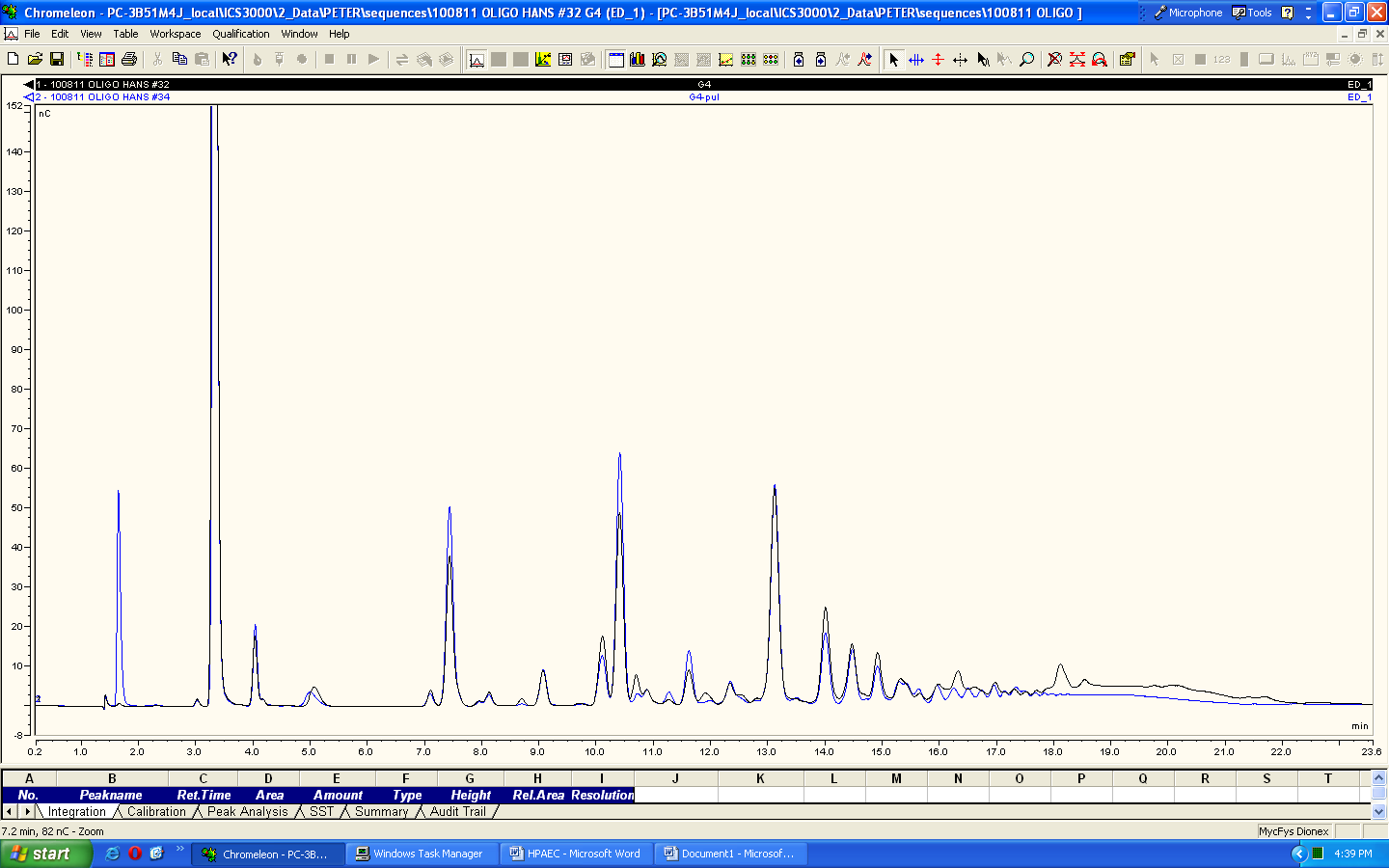


**Fig. S5** HPAEC analysis of the α-glucan products of 4,6-αGT-W from maltotetraose (G4) (black line) and after (blue line) incubation with pullulanase type M1. Pullulanase type M1 hydrolyzes α1→6 glycosidic linkages at the reducing side of an α1→4 glycosidic linkage. Pullulanase hydrolysis would thus demonstrate that 4,6-αGT-W catalyzes α1→4 elongation in addition to α1→6 elongation, its main activity. The comparison shows that some of the larger products, eluting after 15 min, are hydrolyzed yielding smaller compounds proofing that 4,6-αGT-W has some α1→4 elongating activity onto non-reducing end glucose moieties linked via α1→6 bonds.

Reference List

Cantarel BL, Coutinho PM, Rancurel C, Bernard T, Lombard V, Henrissat B (2009) The Carbohydrate-Active EnZymes database (CAZy): an expert resource for glycogenomics. Nucleic Acids Res 37:D233-D238

Dobruchowska JM, Gerwig GJ, Kralj S, Grijpstra P, Leemhuis H, Dijkhuizen L, Kamerling JP (2012) Structural characterization of linear isomalto/malto-oligomer products synthesized by the novel GTFB 4,6-α-glucanotransferase enzyme from *Lactobacillus reuteri* 121. Glycobiology doi: 10.1093/glycob/cwr167:
